# Enhanced Methylene Blue and Diclofenac Removal in Ho/Ga Codoped ZnO: Balancing Defect Chemistry, Surface Accessibility, and Interfacial Oxidation

**DOI:** 10.1021/acsomega.6c04116

**Published:** 2026-08-18

**Authors:** Yonny Romaguera-Barcelay, Ellyson S. Paula-Alves, Aimée G. Jerônimo, Willams A. S. Albuquerque, Luciano C. Almeida, Ramón R. Peña-Garcia

**Affiliations:** † 67744 Universidade Federal Rural de Pernambuco, Programa de pós-graduação em Engenharia Física, Unidade Acadêmica do Cabo de Santo Agostinho, Cabo de Santo Agostinho, Pernambuco 54518-430, Brasil; ‡ Universidade Federal de Piauí, Pós-graduação em Ciências e Engenharia dos Materiais, Teresina, Piauí 64049-550, Brasil; § Universidade Federal de Pernambuco, Departamento de Engenharia Química, Recife, Pernambuco 50740-521, Brasil

## Abstract

Developing ZnO-based photocatalysts for organic-pollutant removal requires materials in which structural modification, surface accessibility, and reactive oxygen species generation are effectively balanced. In this work, Ho/Ga codoped ZnO powders were evaluated as photocatalysts for the removal of methylene blue (MB) and diclofenac (DCF), selected as representative dye and pharmaceutical contaminants with different interfacial behaviors. Zn_1–x–y_Ho_x_Ga_y_O powders with (x, y) = (0.00, 0.00), (0.005, 0.015), and (0.02, 0.03) were synthesized by coprecipitation and calcined at 450 °C. The structural, textural, morphological, and optical responses were correlated with photocatalytic performance, reactive species formation, and reuse stability to clarify how Ho/Ga addition influences the activity of ZnO. All samples preserved the wurtzite ZnO structure, while codoping refined the crystallite size and modified the accessible surface area and defect-related optical response. Among the investigated compositions, Zn_0.98_Ho_0.005_Ga_0.015_O showed the best photocatalytic response, with removal efficiencies of 86% for MB and 70% for DCF after 120 min, and apparent rate constants of 0.0161 and 0.00956 min^–1^, respectively. Scavenger tests showed that hydroxyl-radical-mediated oxidation was the most strongly affected pathway, while oxygen-reduction-related and hole-mediated steps made secondary but relevant contributions depending on the contaminant. The comparison between MB and DCF showed that pollutant-dependent interfacial behavior affects how the same catalyst promotes surface-mediated discoloration and solution-driven oxidative conversion. The improved performance of Zn_0.98_Ho_0.005_Ga_0.015_O is associated with its more favorable defect-related optical response and accessible surface configuration, which support oxidative conversion without the stronger lattice disorder observed at higher codoping levels. Reuse tests showed higher stability for MB than for DCF, with the activity decreasing from 86% to 83% after three cycles for MB and from 70% to 53% after four cycles for DCF, suggesting stronger interfacial deactivation during DCF removal.

## Graphical abstract

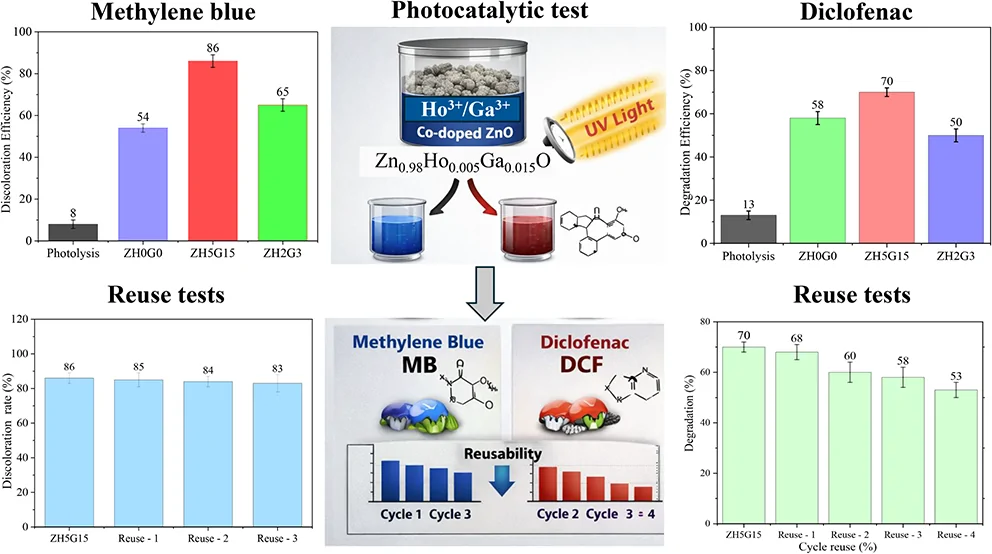

## 1 Introduction

Zinc oxide (ZnO) is a semiconductor whose photocatalytic response depends on the combined influence of electronic structure, defect chemistry, and surface characteristics, rather than on light absorption alone.^1,2^ Defect-related states within the band gap play a central role in modulating charge separation, recombination kinetics, and interfacial redox processes.^1,3^ As a result, modulating defect-related states is a critical aspect of ZnO-based photocatalysis, since these states influence charge utilization without necessarily increasing recombination pathways.^1,3^ This aspect is particularly relevant in heterogeneous photocatalysis, where the final activity is determined by the coupling between charge generation, charge transport, and interfacial reactions involving reactive oxygen species.

Aliovalent doping has been widely used to tune ZnO photocatalysts because it can simultaneously modify lattice organization, defect equilibria, carrier behavior, and the interfacial redox environment.^2^ Among trivalent dopants, Ga^3+^ is commonly associated with donor-type electronic modulation in ZnO, affecting conductivity, crystallinity, and defect-related states.^4,5^ By contrast, rare-earth ions usually introduce a different type of perturbation, more closely related to localized structural distortion and defect-assisted electronic redistribution than to purely donor behavior.^6,7^ Ho^3+^ was selected as the rare-earth codopant because its size and coordination mismatch relative to tetrahedral Zn^2+^ , together with its shielded 4f electronic configuration, can promote local lattice distortion and defect-assisted optical redistribution while preserving the ZnO framework.^7,8^ In this context, Ho^3+^ incorporation is expected to modify defect-assisted recombination pathways and influence the redistribution of photogenerated carriers during photocatalysis. Although the individual effects of Ga^3+^ and Ho^3+^ on ZnO have been explored separately, their combined influence remains poorly understood, particularly when the central question is not only how the defect population changes but also how these changes translate into photocatalytic response under different oxidation regimes. In this sense, codoping offers a route to jointly modulate charge compensation, lattice distortion, and defect-mediated interfacial processes within a single material system.^3,9^ Recent studies on modified and codoped oxide-based photocatalysts, including ferrite-, titanate-, and perovskite-derived systems, have further shown that combining compositional modulation with heterointerfacial design can improve the degradation of pharmaceutical and dye pollutants by altering light absorption, charge utilization, surface accessibility, and ROS-mediated pathways.^10–14^ However, the photocatalytic response does not necessarily improve as the dopant content increases, indicating that performance depends on how charge transport, defect redistribution, and surface accessibility evolve simultaneously.

Synthesis conditions also play a decisive role in determining the final defect structure and surface reactivity of ZnO.^8,15^ In coprecipitation routes, parameters such as precipitation pH influence hydrolysis, nucleation, dopant incorporation, and precursor evolution, thereby affecting crystallite size, microstrain, and accessible surface area after calcination.^8,15^ Calcination temperature further controls the balance between crystallinity and defect retention, which is especially relevant in photocatalytic systems where structural order must coexist with a sufficient population of active defect sites.^15^ Thus, composition and synthesis conditions must be considered together when evaluating the photocatalytic behavior of doped ZnO systems.

A common limitation in the evaluation of ZnO-based photocatalysts, particularly in doped and codoped systems, is the reliance on a single model contaminant, which does not fully capture the diversity of interfacial processes governing pollutant removal.^16^ This limitation becomes even more critical when the defect structure is intentionally modified, because changes in defect distribution and charge-carrier dynamics may not affect all reaction pathways equally. Under these conditions, the choice of probe molecules is not merely operational but essential for determining whether a given photocatalyst preferentially enhances surface-mediated removal, liquid-phase oxidation, or overall reactive oxygen species generation.

Methylene blue (MB) and diclofenac (DCF) are considered here as two systems with distinct interfacial behavior.^16–18^ The selection of MB and DCF was therefore intended to probe two distinct photocatalytic regimes within the same catalyst series. MB is a cationic dye whose removal is strongly influenced by surface interactions and chromophore disruption,^16^ whereas DCF, which is predominantly present in an anionic form under typical aqueous conditions, shows limited adsorption, and its removal depends mainly on oxidation processes occurring in the liquid phase.^17–19^ The use of these two molecules as complementary probe systems, therefore, enables the distinction between surface-influenced and more solution-driven oxidation regimes, allowing the photocatalytic consequences of codoping to be evaluated beyond a single-contaminant response.

Although Ga^3+^ - and Ho^3+^-modified ZnO systems have been individually explored, their combined incorporation remains comparatively less addressed, particularly regarding how donor-type electronic modulation and rare-earth-induced local lattice distortion jointly affect photocatalytic behavior under distinct reaction regimes.^4,8^ This point is relevant because codoping does not necessarily produce a monotonic improvement in photocatalytic activity; instead, the final response depends on the balance between lattice perturbation, defect redistribution, charge-carrier utilization, and surface accessibility.^3,20^ The simultaneous incorporation of Ho^3+^ and Ga^3+^ was used to couple donor-type electronic modulation with rare-earth-induced local lattice distortion within the same ZnO matrix. This design makes it possible to evaluate how these perturbations jointly affect charge utilization, defect-related optical response, and interfacial oxidation during photocatalysis.^4,8^

In codoped ZnO systems, phase stability and morphology evolution provide useful structural information, but they do not fully describe how the material operates during photocatalysis. A more complete assessment requires connecting compositional changes with charge-carrier behavior, surface availability, reactive-species formation, and pollutant-dependent oxidation pathways. The use of MB and DCF as complementary probe molecules further extends this analysis because each contaminant emphasizes a different aspect of the photocatalytic process: MB is more sensitive to surface-influenced discoloration and chromophore oxidation, whereas DCF probes a more solution-driven oxidative pathway with limited adsorption. This comparison allows the composition-dependent activity to be evaluated not only from structural and optical changes, but also from ROS generation, kinetic response, and reuse stability. This approach provides a basis for assessing how the codoping level controls the conversion of structural and defect-related changes into photocatalytic response under different oxidative demands. Based on these considerations, this study investigates Zn_1–x–y_Ho_x_Ga_y_O powders synthesized by coprecipitation at pH 9 and calcined at 450 °C, with emphasis on how the combined incorporation of Ho^3+^ and Ga^3+^ affects the photocatalytic response of ZnO when probed under two distinct oxidative regimes. The selected compositions, (x, y) = (0.00, 0.00), (0.005, 0.015), and (0.02, 0.03), were chosen to compare three clearly differentiated Ho/Ga incorporation regimes: undoped ZnO, moderate codoping, and higher codoping. The compositional interval between the two codoped samples was intentionally kept broad to separate a regime in which donor-type electronic modulation and rare-earth-induced lattice perturbation are expected to remain moderate from a regime in which stronger codopant incorporation may intensify microstructural disorder, defect redistribution, and surface restructuring. This contrast emphasizes the transition from controlled Ho/Ga incorporation to a more strongly perturbed lattice and surface configuration, allowing the photocatalytic response to be related to different levels of codopant-induced structural, optical, and interfacial modification under the oxidative demands imposed by MB and DCF. By comparing methylene blue and diclofenac as probe molecules and correlating their photocatalytic removal behavior with the structural, textural, and optical characteristics of the powders, this study aims to clarify how the Ho/Ga codoping level governs the photocatalytic response of ZnO under distinct oxidative regimes.

## 2 Materials and Methods

### 2.1 Materials

Zinc nitrate hexahydrate (Zn­(NO_3_)_2_·6H_2_O, 98.99%, Neon Commercial, Brazil), holmium­(III) nitrate pentahydrate (Ho­(NO_3_)_3_·5H_2_O, 99.99%, Sigma-Aldrich, Brazil), and gallium­(III) nitrate hydrate (Ga­(NO_3_)_3_·xH_2_O, 99.99%, Sigma-Aldrich, Brazil) were used as precursor salts for the preparation of Zn_1–x–y_Ho_x_Ga_y_O powders. Distilled water and ethanol (99.5%) were used as solvent media. Ethanol was also employed during the washing steps to remove residual ionic species and soluble byproducts. The pH was adjusted with aqueous ammonium hydroxide (NH_4_OH). All chemicals were used as received, without further purification.

### 2.2 Synthesis of Zn_1–x–y_Ho_x_Ga_y_O Powders

The Zn_1–x–y_Ho_x_Ga_y_O powders were synthesized by a coprecipitation route adapted for Ho/Ga-modified ZnO systems. Three nominal compositions were obtained, corresponding to (x, y) = (0.00, 0.00), (0.005, 0.015), and (0.02, 0.03), hereafter denoted as ZH0G0, ZH5G15, and ZH2G3, respectively. Stoichiometric amounts of the nitrate precursors were dissolved at room temperature under magnetic stirring in 100 mL of an ethanol/distilled water mixture (80/20 vol%). The pH was adjusted to 9 by dropwise addition of NH_4_OH to promote precipitation. The resulting precipitates were separated by centrifugation, washed five times with ethanol, dried at 100 °C for 12 h, and calcined at 450 °C for 3 h to obtain nanocrystalline powders.

### 2.3 Material Characterization

The powders were investigated by X-ray diffraction (XRD) using a Bruker D8 Advance diffractometer with Cu Kα radiation (λ = 1.5406 Å). Raman measurements were carried out using a Horiba Xplora spectrometer employing a 532 nm laser operated at 10 mW. The surface morphology of the samples was examined by scanning electron microscopy (SEM) using a TESCAN VEGA3 microscope operated at 15 kV and equipped with an energy-dispersive spectroscopy (EDS) detector for elemental analysis. Photoluminescence (PL) spectra were obtained at room temperature using a Horiba Jobin-Yvon Fluorolog-3 spectrofluorometer, employing an excitation wavelength of 340 nm and excitation/emission slit widths of 5 nm. Diffuse reflectance spectra were collected using a Shimadzu UV-2700 spectrophotometer fitted with an integrating sphere.

### 2.4 Photocatalytic Experiments

The photocatalytic activity of ZH0G0, ZH5G15, and ZH2G3 was evaluated using diclofenac (DCF) and methylene blue (MB) as model contaminants. For each experiment, 0.05 g of photocatalyst was dispersed in 100 mL of aqueous solution containing DCF (10 mg L^–1^) or MB (20 mg L^–1^). The photocatalytic tests were carried out at the natural initial pH of each suspension, allowing the samples to be compared under the same experimental conditions within each pollutant system.

Before irradiation, the suspensions were magnetically stirred in the dark for 30 min to allow the catalyst–pollutant system to approach adsorption–desorption equilibrium before photocatalysis. The concentration measured immediately after this dark-equilibration step was taken as C_0_ for the kinetic and efficiency calculations, allowing the irradiation profiles to be interpreted from the postadsorption state and distinguishing photocatalytic removal from the initial dark uptake by the catalyst. The mixtures were then exposed to a 125 W UV-emitting commercial lamp with the outer bulb removed. According to the manufacturer, the lamp emits in the 350–450 nm range, and the irradiance at the sample position was 6.0 ± 0.2 mW cm^–2^. This irradiation configuration was used to activate the ZnO-based photocatalysts under controlled UV illumination, and photolysis experiments were performed under the same conditions to distinguish direct photochemical removal from catalyst-assisted removal. Aliquots were withdrawn after 0, 10, 20, 30, 45, 60, 90, and 120 min of irradiation. The residual concentration of DCF and MB was monitored by UV–Vis spectroscopy at 280 and 664 nm, respectively, and the corresponding absorbance decay was used to estimate the photocatalytic removal efficiency of the target molecules. The temporal decay of these diagnostic absorption bands was used to calculate C/C_0_ and the corresponding removal efficiencies, which refer to the monitored UV–Vis signals under the present experimental conditions. The removal profiles were expressed as normalized concentration values (C/C_0_), where C_0_ is the concentration measured after the dark equilibration step, and C is the concentration at irradiation time t. The removal efficiency was calculated as Removal (%) = [(C_0_–C)/C_0_] × 100. Radical-scavenging experiments were performed under identical photocatalytic conditions to evaluate the oxidative pathways involved in the removal of DCF and MB. After the 30 min dark-equilibration step, EDTA, methanol, and chloroform were introduced into the suspension as commonly used molecular probes for hole-mediated (h^+^), hydroxyl-radical-mediated (•OH), and oxygen-reduction-related pathways, respectively.^21,22^ Samples were collected at the beginning and after 120 min of irradiation, and the inhibition induced by each probe was compared with the corresponding scavenger-free experiment. Reusability tests were performed under the same photocatalytic conditions described above. After each cycle, the catalyst was recovered by centrifugation, washed with distilled water to remove weakly adsorbed reaction residues, dried at 100 °C, and weighed before the next run. Each subsequent cycle was carried out using a fresh pollutant solution, and the experimental volume was adjusted according to the recovered catalyst mass to maintain the same catalyst loading used in the initial experiment. No fresh catalyst was added between cycles.

## 3 Results and Discussion

### 3.1 Structural Analysis

Figure 1 presents the XRD patterns of ZH0G0, ZH5G15, and ZH2G3 together with their corresponding Rietveld refinements. The calculated profiles reproduce the experimental peak positions and line shapes with small residuals over the measured 2θ range, and the difference curves do not show marked systematic deviations. The refinement indicators listed in Table 1, particularly the GOF values between 1.15 and 1.27 and the comparable Rp/Rwp values, support the internal consistency of the structural model used to analyze the series.^23^ All reflections can be indexed to hexagonal zincite ZnO (*P*6_3_mc), consistent with the reported structural parameters of wurtzite ZnO.^24^ No additional reflections attributable to crystalline Ho_2_O_3_ or Ga_2_O_3_ secondary phases are detected in Figure 1. The persistence of the wurtzite pattern across the investigated compositions is consistent with previous reports on Ga-doped and trivalent codoped ZnO systems, in which structural evolution occurs predominantly through line broadening, lattice perturbation, and microstructural modification rather than through detectable crystalline segregation.^25,26^ The refined lattice metrics (Table 1) reveal a small but systematic contraction of the average unit cell with increasing nominal codopant loading. Relative to ZH0G0, ZH5G15 shows a slight contraction, whereas ZH2G3 exhibits a clearer decrease in both *a* and *c*. Specifically, the lattice parameter a decreases from 3.2518(5) Å to 3.2441(1) Å (−0.24%), while c decreases from 5.2088(1) Å to 5.2022(4) Å (−0.13%), leading to a reduction in unit-cell volume from 47.6987 Å^3^ to 47.4093 Å^3^ (−0.61%) (Table 1).

**Figure 1:**
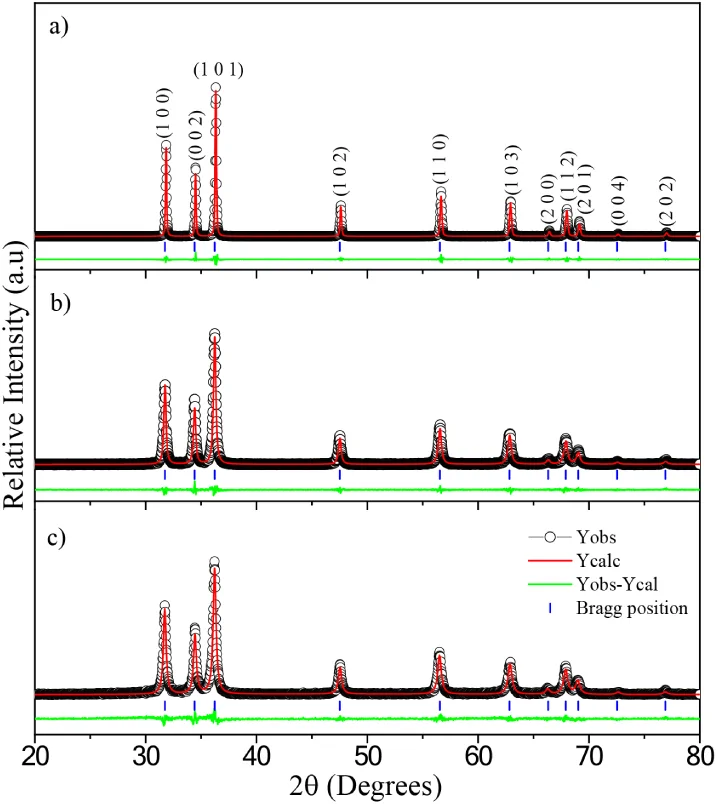
X-ray diffraction patterns and corresponding Rietveld refinements of Zn_1–x–y_Ho_x_Ga_y_O powders: a) ZH0G0, b) ZH5G15, and c) ZH2G3. Experimental data (black), calculated profiles (red), and difference curves (Yobs—Ycalc, green) are shown together with the Bragg positions of the wurtzite ZnO phase (*P*6_3_mc).

**Table 1:** Rietveld Refinement Results for Zn_1–x–y_Ho_x_Ga_y_O Powders (ZH0G0, ZH5G15, and ZH2G3), Including Agreement Factors, Phase Fraction, Lattice Parameters, Unit-Cell Volume, Crystallite Size, and Microstrain^a^

Parameters	ref 24	ZH0G0	ZH5G15	ZH2G3
Rexp (%)	-	14.16	13.62	15.16
Rwp (%)	-	17.64	15.67	19.28
Rp (%)	-	10.01	9.55	12.84
GOF	-	1.25	1.15	1.27
R-Bragg	-	1.627	1.35	2.201
ZnO (P63mc)	-	100	100	100
*a* (Å)	3.250*	3.2518(5)	3.2501(3)	3.2441(1)
*c* (Å)	5.207*	5.2088(1)	5.2034(3)	5.2022(4)
Volume (Å^3^)	47.63*	47.6987	47.5982	47.4093
Crystallite size (nm)	-	100.5(2)	49.4(2)	28.8(2)
Strain	-	0.01	0.24(2)	0.27(3)

a Reference values for wurtzite ZnO are provided for comparison.^24^

Although the magnitude of this variation is modest, its progressive character indicates a composition-dependent average lattice response. The monotonic contraction is more consistent with the contribution of Ga^3+^ (0.47 Å) in tetrahedrally coordinated environments, given its smaller ionic radius relative to Zn^2 +^ (0.60 Å) in the same coordination.^27^ By contrast, the structural role of Ho^3+^ (0.901 Å) is less straightforward, since its effective ionic radius depends strongly on coordination; therefore, its contribution is more likely reflected in local lattice perturbation and strain fields rather than as a direct driver of average cell expansion or contraction.^27^ The microstructural response is more pronounced than the shift in the average lattice parameters.

Table 1 shows a marked decrease in coherent domain size with increasing nominal dopant content, from 100.5(2) nm for ZH0G0 to 49.4(2) nm for ZH5G15 and 28.8(2) nm for ZH2G3. This reduction indicates that increasing aliovalent content restricts crystallite growth during calcination at 450 °C. The strain values reported in Table 1 also indicate a progressively more distorted lattice in the codoped samples, increasing from 0.24(2) for ZH5G15 to 0.27(3) for ZH2G3. Accordingly, the principal structural response to codoping is expressed more strongly in the defect-sensitive microstructural parameters than in the average lattice metrics. Such behavior is compatible with dopant-induced local distortion and charge-compensation-related defect fields, which hinder defect-free crystal growth while preserving the crystallographic framework of ZnO.^25,26^

Figure 2 shows the room-temperature Raman spectra of ZH0G0, ZH5G15, and ZH2G3 samples. Table 2 summarizes the peak positions and fitted band areas. The spectra were normalized, and the Raman bands were fitted using oscillator functions after baseline correction (Figure S1a–c).^28^ ZH0G0 exhibits the Raman response expected for wurtzite ZnO, with bands assigned to E_2_^(low)^ at ∼97 cm^–1^, E_2_^(high)^ at 428 cm^–1^, and higher-order features such as 2E_2_^(low)^ and E_2_^(high)^ – E_2_^(low)^, in agreement with the reported vibrational pattern of ZnO.^29,30^ In this spectrum, the presence of the E_2_-type modes together with the absence of additional symmetry-related bands is consistent with the phase assignment obtained from XRD.^29^ Upon Ho^3+^/Ga^3+^ codoping, the E_2_^(high)^ mode shifts to a higher wavenumber and becomes broader. Relative to ZH0G0, this band moves to 435 cm^–1^ in ZH5G15 and 434 cm^–1^ in ZH2G3, while its broader line shape indicates a wider distribution of local vibrational environments (Figure 2, Table 2). In ZnO, the E_2_^(high)^ mode is particularly sensitive to short-range lattice perturbation because it primarily involves oxygen sublattice vibrations.

**Figure 2:**
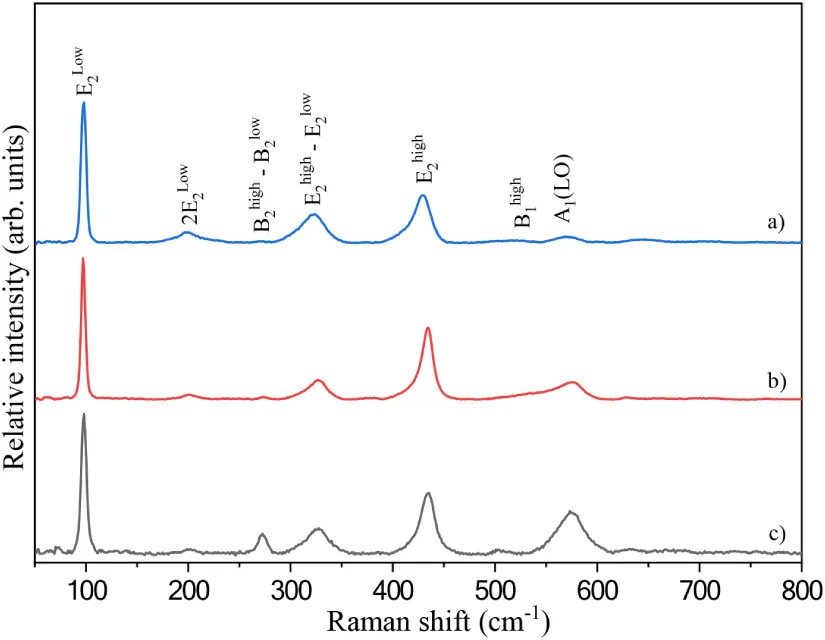
Raman spectra of Zn_1–x–y_Ho_x_Ga_y_O powders: a) ZH0G0, b) ZH5G15, and c) ZH2G3, corresponding to (x, y) = (0.00, 0.00), (0.005, 0.015), and (0.020, 0.030), respectively. The characteristic vibrational modes of wurtzite ZnO are indicated.

**Table 2:** Raman Peak Positions and Corresponding Integrated Peak Areas for ZH0G0, ZH5G15, and ZH2G3^a^

Assignment	ZnO [30]	ZH0G0	ZH5G15	ZH2G3
E_2_^low^ Peak area	99	97(320)	99(300)	98(333)
2E_2_^low^	206	199	203	202
B_1_^high^ – B_1_^low^	284	270	274	272
E_2_^high^ – E_2_^low^ Peak area	330	321(670)	327(425)	327(512)
A_1_ (TO)	378		378	382
E_1_ (TO)	411	410	410	415
E_2_^high^ Peak area	435	428(84)	435(902)	434(890)
B_1_ ^high^	525	517	546(260)	560(210)
A_1_ (LO) Peak area	580	570(158)	576(450)	577(720)
E_1_ (LO)	618	649	633	632
LA + TO	700	707		677

a Mode assignments were performed according to the characteristic Raman-active phonon modes of wurtzite ZnO reported in the literature.^30,34^ Peak areas were determined after baseline correction using oscillator-function fitting in Igor Pro software, allowing consistent quantification of the vibrational contributions across the series.

The upshift observed after codoping therefore suggests a modification of the local restoring forces acting on the ZnO lattice, whereas the concomitant broadening is consistent with increased structural heterogeneity at the local scale. This type of response has been observed in Ga-doped ZnO, where aliovalent substitution perturbs the phonon line shape due to local disorder and defect-assisted scattering.^31,32^ A similar behavior has also been observed in Ho-doped ZnO systems, where the wurtzite structure is maintained, but the local vibrational response becomes less uniform.^33^ This Raman evolution is consistent with the XRD/Rietveld results. While diffraction indicated only a modest contraction of the average unit cell, it also showed a marked reduction in coherent domain size and higher strain in the codoped powders. Raman spectroscopy resolves this structural response at a more local level: the average crystallographic symmetry remains unchanged, but the vibrational signature becomes increasingly sensitive to bond-length fluctuations, lattice distortion, and defect-related perturbation.

As the spectra were normalized, the ratio I­(LO)/I­(E_2_^(high)^), defined here as the fitted A_1_(LO) area divided by the fitted E_2_^(high)^ area, was used only as a comparative descriptor of defect-sensitive LO-related scattering (Supporting Information, Figure S1a **c**). This point is important because LO-related modes are more sensitive to deviations from ideal lattice periodicity than the nonpolar E_2_(high) mode. The A_1_(LO) band in the 570–577 cm^–1^ range becomes more prominent after codoping, and its fitted area increases from 158 au for ZH0G0 to 450 au for ZH5G15 and 720 au for ZH2G3 (Table 2). In ZnO-based systems, LO-related scattering is commonly enhanced by deviation from ideal lattice periodicity, since point defects, local strain, and compositional heterogeneity relax the Raman selection rules and increase the contribution of defect-assisted vibrational processes.^30,31,34^

The stronger LO response observed after codoping is therefore consistent with a larger population of locally perturbed environments, even though the average wurtzite framework remains unchanged. At the same time, the normalized ratio I­(LO)/I­(E_2_^(high)^) changes from 1.88 in ZH0G0 to 0.50 in ZH5G15 and 0.81 in ZH2G3, indicating that the evolution of LO-related scattering is not strictly monotonic when referenced to the E_2_^(high)^ contribution. This point is important because the spectra were normalized before fitting; accordingly, this ratio reflects the relative redistribution of spectral weight between defect-sensitive and lattice-sensitive modes, rather than a direct measure of defect concentration. An additional change is observed in the higher wavenumber LO-related band, which shifts from 649 cm^–1^ in ZH0G0 to ∼633 cm^–1^ in ZH5G15 and ∼632 cm^–1^ in ZH2G3. This displacement further indicates that codoping modifies the local vibrational landscape of the ZnO lattice, affecting not only the intensity of disorder-activated features but also the energetic position of LO-related modes.^30,31,34^

### 3.2 Surface Morphology, Elemental Composition, and Textural Properties

Figure 3a–c show the SEM micrographs of ZH0G0, ZH5G15, and ZH2G3, while Figure 3d–f display the corresponding merged EDS elemental maps. All samples are composed of strongly agglomerated particles with irregular surfaces and visible interagglomerate voids. At this scale, the powder is organized as secondary agglomerates formed by finely divided oxide particles that remain strongly associated after precipitation and thermal treatment.^35^ ZH0G0 (Figure 3a) consists of agglomerates made from more rounded and clearly defined subunits, leading to a comparatively looser texture where the interagglomerate voids are more easily discernible. By contrast, ZH5G15 and ZH2G3 (Figure 3b, c) display a finer and more compact agglomerated texture, with less individualized constituent units and a less open void network. This morphological evolution indicates that Ho/Ga addition modifies the texture of the agglomerated powder and leads to a more compact arrangement after calcination.

**Figure 3:**
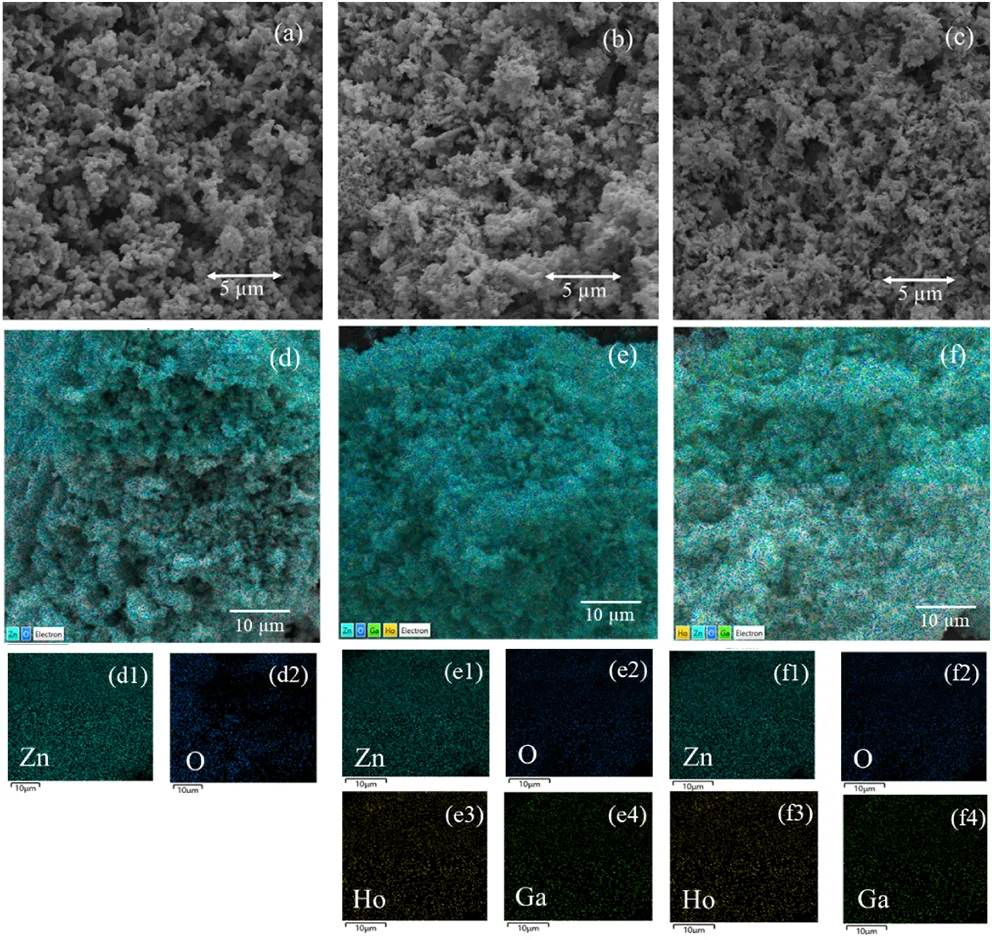
SEM micrographs of (a) ZH0G0, (b) ZH5G15, and (c) ZH2G3, showing the morphological changes associated with Ho/Ga addition. Corresponding merged EDS elemental maps are shown for (d) ZH0G0, (e) ZH5G15, and (f) ZH2G3. Individual Zn and O maps are presented in (d1, d2), whereas Zn, O, Ho, and Ga maps are shown in (e1–e4) and (f1–f4) for the codoped samples. Scale bars: 5 μm for SEM images and 10 μm for EDS maps.

Accordingly, codoping does not suppress aggregation but modifies the organization of the secondary particles, producing a finer agglomerated texture.^36,37^ Similar morphology changes have been reported for doped ZnO powders obtained by precipitation-based routes.^38^ This morphological refinement agrees with the microstructural evolution obtained from XRD, where Ho/Ga addition led to a progressive reduction in coherent domain size and an increase in lattice strain. Although SEM and XRD probe different length scales, the combined results show that codoping modifies the crystallization pathway during calcination, restricting the growth of crystalline domains while promoting a finer and more compact agglomerated texture. To complement the morphological analysis, EDS elemental mapping was used to provide qualitative compositional information from the regions examined by SEM. ZH0G0 shows only the expected Zn and O distributions, with the individual maps presented in Figure 3(d, d1, and d2), whereas ZH5G15 and ZH2G3 additionally show Ho- and Ga-related map contrasts, as displayed in Figure 3(e, e1–e4) and 3(f, f1–f4), respectively.

These maps indicate the spatial distribution of the codopant-related signals within the analyzed regions. The correspondence between the dopant-related maps and the ZnO agglomerated regions indicates that Ho and Ga are spatially associated with the oxide particles, with no evident formation of isolated dopant-rich domains at the SEM/EDS observation scale. The corresponding EDS spectra are provided in Figure S2 as complementary compositional information. The Au signal originates from the conductive coating used during SEM/EDS analysis, and no unexpected elemental contributions, apart from those associated with the prepared compositions and the coating layer, are detected.^39,40^

The textural consequences of this morphological organization are reflected in the N_2_ adsorption–desorption behavior shown in Figure 4, while Table 3 summarizes the Brunauer–Emmett–Teller (BET) specific surface area, pore volume, and Barrett–Joyner–Halenda (BJH) pore size distribution. All samples show low adsorption at low relative pressure, followed by a pronounced uptake as P/P_0_ approaches 1, indicating that the accessible porosity is dominated by voids associated with the packing of the agglomerated particles, rather than by a significant microporous contribution.^37^ The BET surface area ranges from 9.3 to 12.4 m^2^ g^–1^ across the series. The intermediate composition ZH5G15 shows the highest value (12.4 m^2^ g^–1^), followed by ZH0G0 (10.1 m^2^ g^–1^) and ZH2G3 (9.3 m^2^ g^–1^). Thus, the specific surface area reaches a maximum at the intermediate composition rather than increasing continuously with codopant loading.

**Figure 4:**
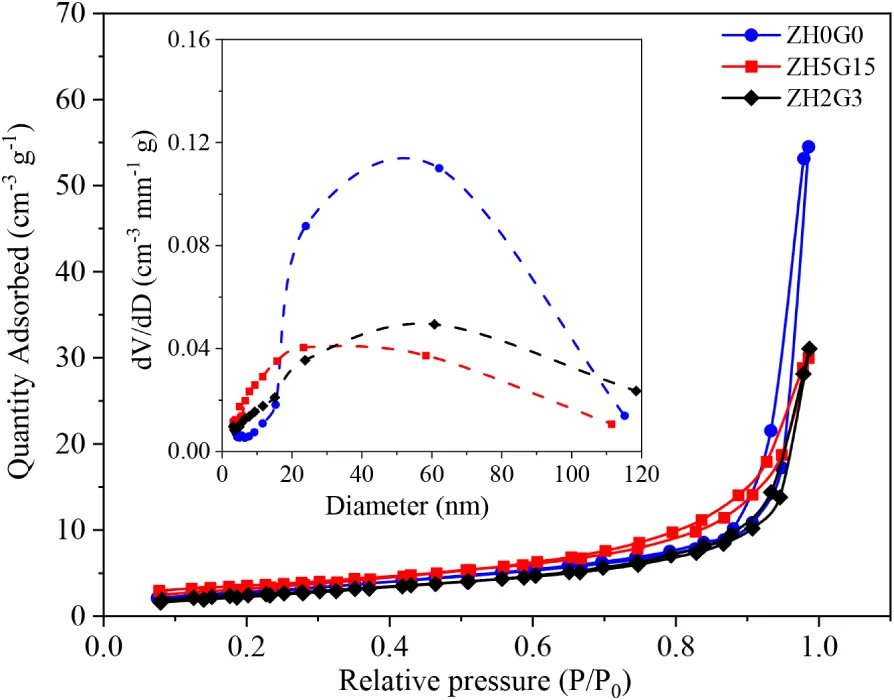
N_2_ adsorption–desorption isotherms of ZH0G0, ZH5G15, and ZH2G3 obtained from N_2_ physisorption measurements, illustrating the textural characteristics of the samples. The inset shows the BJH pore-size distribution, evidencing that Ho^3+^ /Ga^3+^ codoping alters the pore architecture and modulates the accessible surface area of the ZnO powders.

**Table 3:** BET Specific Surface Area and BJH-Derived Pore Parameters of ZH0G0, ZH5G15, and ZH2G3 Samples

	Surface area m^2^ g^–1^ BET method	Pore volume cm^3^ g^–1^ BJH method	Average pore diameter (nm) BJH method
ZH0G0	10.1	0.084	32.1
ZH5G15	12.4	0.046	25.5
ZH2G3	9.3	0.047	32.9

These values are in the same order of magnitude as those reported for Ga-doped ZnO prepared by solution-based methods, where the accessible surface area is strongly affected by dopant concentration and particle-growth behavior.^37,41^ According to the XRD results, the coherent domain size decreases from about 101 nm in ZH0G0 to 49 nm in ZH5G15 and 29 nm in ZH2G3. This refinement contributes to increasing the available surface, but the BET response also depends on how these domains are assembled into secondary agglomerates and on how the interparticle void network remains accessible to N_2_.

In ZH5G15, moderate Ho/Ga addition produces a finer agglomerated texture together with the smallest average BJH pore diameter, favoring a larger accessible interparticle surface and leading to the highest BET area. In ZH2G3, the further decrease in coherent domain size is accompanied by stronger agglomerate compactness, as observed by SEM, which limits the contribution of interparticle voids to the measured surface area. Thus, the maximum surface area at the intermediate composition reflects the balance between crystallite refinement and agglomerate packing, rather than a simple dependence on coherent domain size. This explains why ZH2G3, despite having the smallest crystallites, does not show the highest BET surface area. A similar decoupling between domain refinement and accessible surface area has been reported for Ga-modified ZnO systems, in which higher dopant incorporation may reduce crystallite size and increase lattice strain without producing a proportional increase in accessible surface area.^42^

The BJH analysis further clarifies the textural evolution. ZH0G0 exhibits the highest pore volume (0.084 cm^3^ g^–1^) and an average BJH pore diameter of 32.1 nm, which is consistent with its more open agglomerated texture. In contrast, ZH5G15 and ZH2G3 show lower pore volumes (0.046 and 0.047 cm^3^ g^–1^, respectively), corresponding to a reduction of about 45% relative to ZH0G0. Despite this decrease in total pore volume, ZH5G15 shows the smallest BJH average pore diameter (25.5 nm), indicating that the void network becomes finer and contributes more effectively to the accessible surface area. A similar effect has been reported for Ga-doped ZnO nanostructures, in which dopant concentration modifies particle growth and the resulting pore characteristics through changes in nucleation and growth during synthesis.^41^

The pore-size distribution curves in the inset of Figure 4 reveal a broad mesoporous network for all samples. ZH0G0 shows a stronger contribution from the larger pore region, whereas ZH5G15 shifts toward smaller pores. ZH2G3 maintains a broad distribution, but without the surface-area advantage observed for ZH5G15. Within this series, ZH5G15 combines the highest surface area with the smallest average pore diameter, whereas further codoping in ZH2G3 preserves broad mesoporosity but not the same level of accessible surface. Although the BET surface areas remain modest compared with highly porous ZnO architectures, the observed differences are relevant within this series because they reflect changes in the accessibility of the interparticle surface generated by Ho/Ga addition.

### 3.3 Optical Properties

Figure 5a shows the photoluminescence spectra of ZH0G0, ZH5G15, and ZH2G3. In all samples, the emission is concentrated mainly in the violet-blue to visible region, while the ultraviolet near-band-edge contribution expected for ZnO does not appear as an isolated narrow characteristic. The most intense portion of the spectrum lies between ∼390 and 520 nm, where ZH5G15 exhibits the strongest response, with a maximum in the ∼400–440 nm interval and a shoulder extending toward ∼500 nm. Within this broader short-wavelength region, the ∼470–520 nm interval is commonly associated in ZnO with radiative processes involving Zn-related defects, donor–acceptor-type transitions, and surface-associated states.^43,44^ Beyond ∼525 nm, the spectral behavior changes: ZH0G0 shows comparatively stronger emission and a broader contribution extending from ∼560 to ∼670 nm, whereas ZH5G15 and ZH2G3 display a markedly weaker long-wavelength tail. In ZnO, emission in this range is frequently discussed in relation to deeper radiative channels commonly associated with oxygen-related centers and their charge states, although overlap among different contributions remains significant.^45,46^ The contrast between the stronger short-wavelength emission of ZH5G15 and the more pronounced long-wavelength contribution of ZH0G0 therefore indicates that Ho/Ga codoping changes the balance between the visible radiative channels rather than uniformly enhancing or suppressing the entire photoluminescence (PL) response.

**Figure 5:**
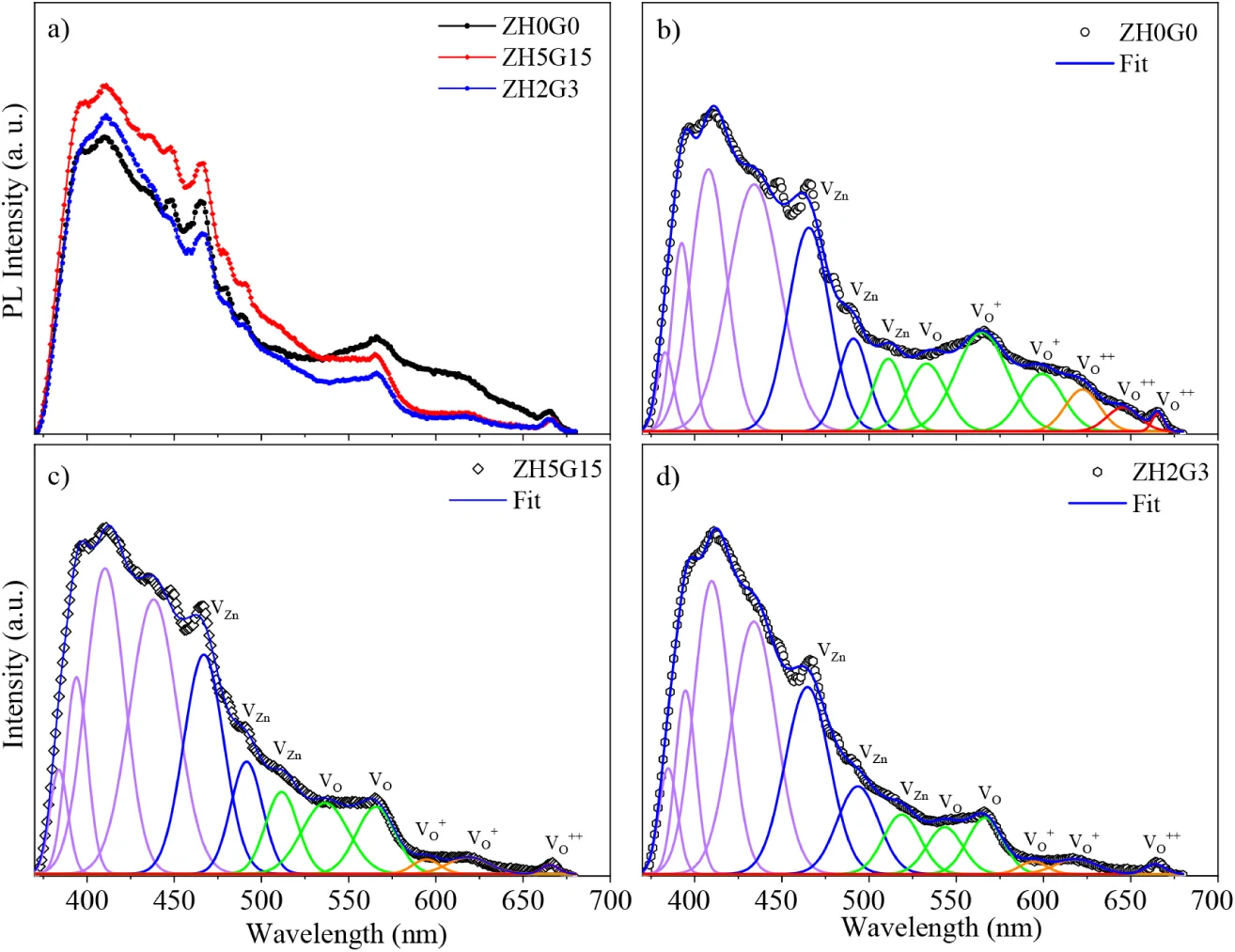
a) Photoluminescence (PL) spectra of ZH0G0, ZH5G15, and ZH2G3, highlighting the effect of Ho^3+^/Ga^3+^ codoping on the emission profile. (b–d) Gaussian deconvolution of the PL spectra for ZH0G0, ZH5G15, and ZH2G3, displaying the individual emission components and the corresponding total fitted curves.

Figure 5b–d and Table 4 show the spectral redistribution more clearly. To compare these spectral changes, the broad PL band was deconvoluted after baseline subtraction using Gaussian components, and the relative contribution of each band was obtained from the integrated area of the fitted peaks normalized to the total fitted emission. In the deconvolution model used here, the 470–520 nm component, typically associated with radiative states related to Zn vacancies and donor–acceptor transitions in ZnO,^43,44,47^ increases in relative area from 25% in ZH0G0 to 29% in ZH5G15 and 31% in ZH2G3. The progressive increase of this contribution after codoping is compatible with the aliovalent character of Ho^3+^ and Ga^3+^, which can modify local charge compensation and favor the relative participation of short-wavelength defect-related radiative channels in the radiative response.^47^ This trend agrees with the higher microstrain observed by XRD and the Raman signatures of increased local lattice perturbation after codoping.

**Table 4:** Gaussian-Deconvoluted PL Components of ZH0G0, ZH5G15, and ZH2G3, Showing the Emission Windows, Common ZnO PL Assignments, and Relative Area Fractions (%)

		Area under curve (%)		
Common ZnO PL assignment	Peaks range (nm)	ZH0G0	ZH5G15	ZH2G3
V_Zn_-related emission	470–520	25	29	31
V_O_-related emission	520–570	5	15	5.4
V_O_^+^-related emission	570–620	10	2	0.3
V_O_^+2^-related emission	620–670	8	2	0.4

A distinct trend is observed for the 520–570 nm component, frequently associated in ZnO with emission commonly linked to oxygen-vacancy-related states. Its relative area increases from 5.0% in ZH0G0 to 15% in ZH5G15 and then decreases to 5.4% in ZH2G3. Thus, unlike the 470–520 nm contribution, this emission does not increase progressively with codoping but reaches a maximum at the intermediate composition. This behavior suggests that moderate Ho/Ga incorporation favors a local compensation regime in which the 520–570 nm oxygen-related radiative contribution becomes more prominent, whereas further codoping increases lattice perturbation and changes the relative balance among overlapping oxygen-related and surface-related contributions to the 520–570 nm emission window.

The most pronounced change is observed in the 570–670 nm region, where ZnO usually exhibits deep visible emission channels commonly associated with oxygen-related centers, emissions often assigned to ionized oxygen-vacancy-related states, and surface-related radiative processes. Within the adopted deconvolution model, the components in this spectral window decrease markedly after codoping. Taken together, the two components in the 570–670 nm range decrease from 18% in ZH0G0 to 4% in ZH5G15 and 0.7% in ZH2G3.

The attenuation of the 570–670 nm contribution suggests that Ho/Ga codoping decreases the relative participation of deep visible radiative channels commonly associated with oxygen-related and surface-related states. Rather than indicating the complete suppression of specific defects, this trend points to a redistribution of defect-assisted recombination pathways within the preserved wurtzite ZnO host. Local charge compensation and lattice perturbation induced by codoping can modify the relative contribution and radiative efficiency of these overlapping defect-related channels, making the shorter-wavelength visible components relatively more prominent in the overall PL response.

This spectral evolution is consistent with trends reported for Ga-modified ZnO^48^ and with reports that associate the orange-red emission of ZnO with oxygen-related radiative processes.^45,46^ In the present series, the PL response reflects a redistribution of overlapping defect-related emission channels rather than the emergence of a new emissive mechanism. This behavior also agrees with the role expected for Ho incorporation, which is more likely to affect the ZnO lattice through local structural perturbation and defect-complex formation than through the formation of an independent radiative phase.^33^ A comparable redistribution of visible PL components has also been reported for trivalent codoped ZnO systems, such as In/Ga-modified ZnO.^36^

The deconvoluted PL components reveal a redistribution of the ZnO defect-related radiative response after Ho/Ga incorporation, with increased contribution from the 470–520 nm region and attenuation of the deeper 570–670 nm visible emission (Table 4). This spectral redistribution is consistent with the Raman evidence of local lattice perturbation and disorder-sensitive vibrational changes, indicating that codoping modifies the relative participation of shallow and deep defect-related optical channels. For comparison, the Gaussian centers obtained from the PL deconvolution were converted into photon energies and positioned within the ZnO band gap range, as shown in Figure S3.

Figure 6 (inset) presents the diffuse-reflectance spectra of ZH0G0, ZH5G15, and ZH2G3. Besides the fundamental absorption edge of ZnO, the Ho-containing samples display narrow bands near ∼454 nm (^5^I_8_ → ^5^G_6_), ∼488 nm (^5^I_8_ → ^3^K_8_), ∼544 nm (^5^I_8_ → ^5^F_4_/^5^S_2_), and ∼650 nm (^5^I_8_ → ^5^F_5_), which are characteristic of intra4f transitions of Ho^3+^.^49,50^ Their appearance only in ZH5G15 and ZH2G3 provides optical evidence of Ho incorporation in the trivalent state. The narrow character of these absorptions is expected, as the *4f* electrons are partially shielded by the outer electron shells. Their greater visibility in the codoped samples is also compatible with a locally perturbed coordination environment, because reduced site symmetry can intensify nominally weak *4f* transitions through parity mixing, as described within the Judd–Ofelt framework.^49,50^

**Figure 6:**
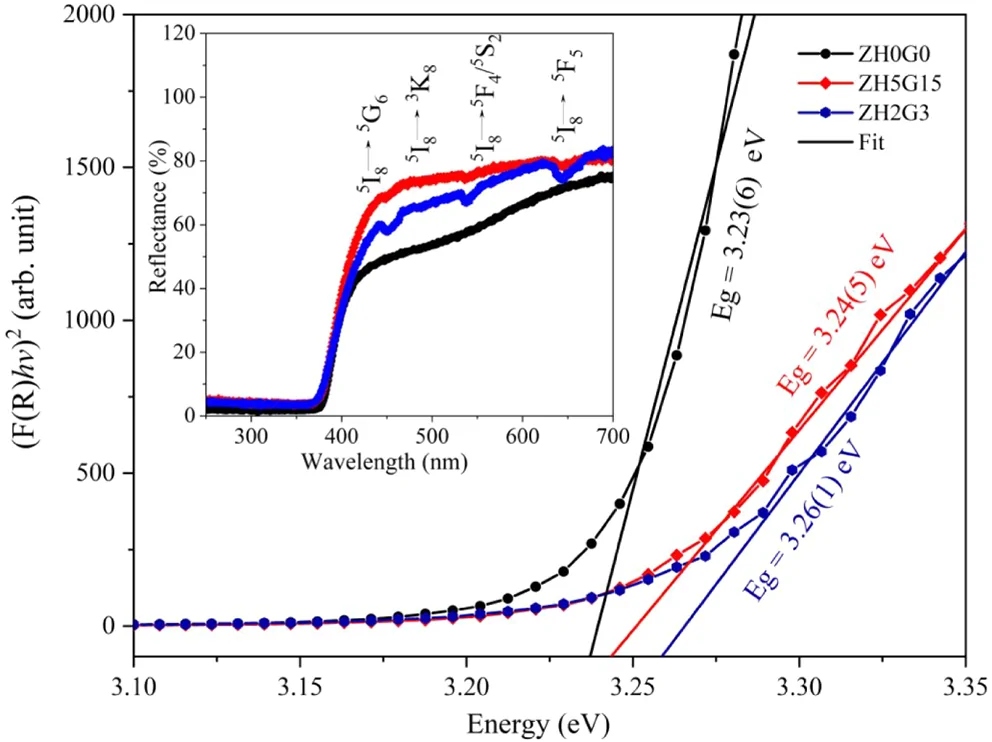
Tauc plots derived from diffuse reflectance spectra (inset) for estimating the apparent optical band gap (*Eg*) of ZH0G0, ZH5G15, and ZH2G3 using the Kubelka–Munk function, assuming a direct electronic transition. The *Eg* values were obtained by extrapolating the linear region of the Tauc plot to the photon-energy axis at (*F*(*R*)*hv*)^2^ = 0.

For the band-edge analysis, the diffuse-reflectance data were converted through the Kubelka–Munk formalism (eq 1), where *R* is the absolute reflectance:^51,52^

1 $$F\left(R\right)=\frac{{\left(1-R\right)}^{2}}{2R}$$

The optical edge was then estimated using the Tauc-type relation (eq 2), appropriate for a direct allowed transition, as commonly adopted for ZnO:^51,52^

2 $${\left(F\left(R\right)hv\right)}^{2}=A\left(hv-E_{g}\right)$$

In this expression, *hν* is the photon energy, *A* is a proportionality constant, and *E*_*g*_ was determined from the intercept of the extrapolated linear portion of the absorption edge with the photon-energy axis, corresponding to (*F*(*R*)*hv*)^2^ = 0. For these strongly scattering and defect-containing powders, the Kubelka–Munk/Tauc-derived *E*_*g*_ is treated as an optical-edge estimate influenced by localized states, subgap absorption, and the selected fitting window.^51,52^

The extrapolated *E*_*g*_ values remained nearly unchanged across the series: 3.23(6) eV for ZH0G0, 3.24(5) eV for ZH5G15, and 3.26(1) eV for ZH2G3. The total variation, approximately 0.03 eV, is comparable to the uncertainty associated with selecting the linear region in Kubelka–Munk/Tauc analysis, indicating that the ZnO band-edge position is essentially preserved after Ho/Ga addition.^51,52^ However, the absorption onset becomes less abrupt in the codoped powders, showing that Ho/Ga incorporation modifies the near-edge absorption profile without producing a comparable displacement of the fundamental ZnO transition.^43–48^ The small *E*_*g*_ variation observed here remains within the range reported for Ga-doped ZnO nanopowders. Depending on dopant content and defect density, Ga-containing ZnO can exhibit either a modest increase in the apparent optical edge or a smaller effective value when band-tail states and subgap absorption become more influential.^26,53^

To quantify this near-edge broadening, the Urbach energy (*E*_*U*_) was estimated from the exponential region of the absorption edge derived from the Kubelka–Munk-transformed diffuse-reflectance spectra, following the methodology commonly used for defect-containing semiconductor powders (Figure S4).^54,55^ The *E*_*U*_ values increased from 36.06 meV for ZH0G0 to 42.96 meV for ZH5G15 and 49.64 meV for ZH2G3, indicating a systematic increase in absorption-tail disorder with codopant loading. Ga^3+^ contributes to charge-compensation effects and donor-type perturbation, whereas Ho^3+^ introduces stronger short-range distortion because of its size and coordination mismatch with the tetrahedral Zn^2+^ environment. The resulting distribution of local strain fields, bond distortions, and defect-associated electronic environments broadens the absorption edge without producing a comparable displacement of the apparent ZnO band gap. The monotonic increase in *E*_*U*_ agrees with the microstrain evolution obtained from Rietveld refinement, the Raman evidence of local vibrational disorder, the Ho^3+^ intra4f absorption bands, and the redistribution of defect-related PL components. Within this series, ZH5G15 occupies an intermediate tail-state regime, while ZH2G3 shows the strongest near-edge energetic disorder.

### 3.4 Photocatalytic Performance

#### 3.4.1 Photocatalytic Discoloration of Methylene Blue

The photocatalytic performance of the Ho/Ga codoped ZnO series was evaluated by comparing two pollutant systems with distinct interfacial behavior. This comparison allows the composition-dependent responses of ZH0G0, ZH5G15, and ZH2G3 to be examined under surface-influenced and more solution-driven oxidative conditions, as typically observed during dye discoloration and pharmaceutical removal processes.^16,56,57^ In addition to the catalyst composition, interfacial photocatalytic processes are influenced by surface charge, pollutant speciation, adsorption affinity, and ROS-mediated oxidation chemistry.^58,59^ Figure 7a shows the UV–Vis spectral evolution of MB in the presence of ZH5G15, selected as the representative catalyst. The characteristic absorption band at 664 nm decreases progressively with irradiation time, while its maximum position remains essentially unchanged. No additional intense band develops in this spectral region during irradiation. This behavior is consistent with photocatalytic discoloration associated with disruption of the conjugated MB chromophore, rather than with the accumulation of new strongly absorbing species in the monitored spectral window. The normalized concentration profiles in Figure 7b, expressed as C/C_0_ as a function of irradiation time, show that the dark stage leads to only limited MB uptake by the catalyst-containing suspensions. In contrast, a much stronger decrease in concentration occurs after UV irradiation. Photolysis remains low throughout the experiment and reaches only ∼8% after 120 min, indicating that direct photochemical bleaching of MB is minor under the present conditions.

**Figure 7:**
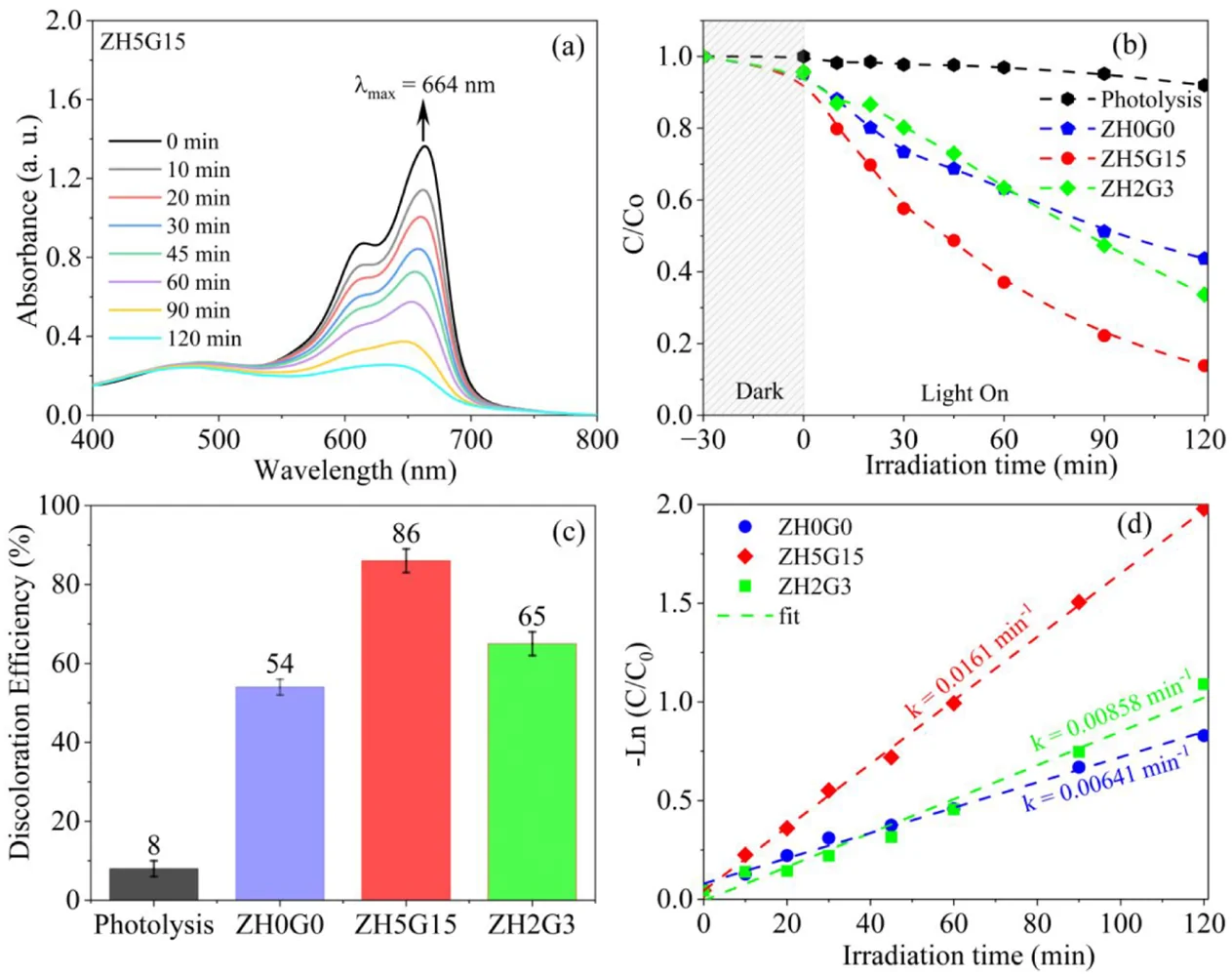
Photocatalytic discoloration of methylene blue (MB). a) UV–Vis spectral evolution of MB (representative example shown for ZH5G15). b) Normalized concentration profiles (C/C_0_) versus irradiation time. c) MB discoloration efficiencies after 120 min for photolysis, ZH0G0, ZH5G15, and ZH2G3. d) Pseudo-first-order kinetic plots (−ln­(C/C_0_) versus time) and fitted rate constants.

Under irradiation, the catalytic responses become clearly differentiated: ZH0G0 reaches 54% MB removal after 120 min, ZH2G3 reaches 65%, and ZH5G15 reaches 86% (Figure 7c). Accordingly, the activity follows the sequence ZH5G15 > ZH2G3 > ZH0G0, with the intermediate composition showing an enhancement of approximately 59% relative to pristine ZnO (86% vs 54%) and about 32% relative to ZH2G3 (86% vs 65%). The kinetic analysis in Figure 7d follows the same ranking. The plots of −ln­(C/C_0_) versus irradiation time are well described by an apparent pseudo-first-order relation under the present dilute-dye conditions, yielding rate constants of 0.00641 min^–1^ for ZH0G0, 0.00858 min^–1^ for ZH2G3, and 0.0161 min^–1^ for ZH5G15. Thus, the intermediate composition is 2.51 times faster than pristine ZnO and 1.88 times faster than ZH2G3. Therefore, the superiority of ZH5G15 is not limited to final conversion but reflects consistently faster reaction kinetics throughout the process.

The photocatalytic ranking is more clearly understood when structural, textural, and optical features are considered together. The photocatalytic trend reflects the combined influence of lattice modification, surface accessibility, and defect-related optical changes induced by Ho/Ga incorporation. ZH5G15 combines smaller crystallites and the highest accessible surface area without the stronger lattice disorder observed for ZH2G3. Although the absolute BET values are not high, the relative increase in accessible surface area observed for ZH5G15 acts in combination with its controlled lattice perturbation and defect-related optical redistribution. This result suggests that the higher MB response of ZH5G15 is associated with increased surface accessibility while avoiding the stronger structural disorder observed for ZH2G3. At the optical level, codoping redistributes the defect-assisted emission, particularly through attenuation of the deepest orange-red channels, while diffuse reflectance reveals a broadened near-edge region without a significant displacement of the optical edge. In ZH5G15, these features are consistent with a defect-related optical response and surface condition more favorable to interfacial oxidation without reaching the stronger local disorder accumulated in ZH2G3. In contrast, the stronger perturbation observed in ZH2G3 does not translate into improved kinetics, indicating that increasing defect-related disorder beyond an intermediate level does not enhance MB discoloration. A similar maximum at intermediate dopant content has been reported for other modified ZnO systems, in which intermediate compositions outperform more heavily doped materials.^41^

To evaluate the oxidative pathways involved in the methylene blue (MB) response observed in Figure 7, scavenger tests were conducted using ZH5G15, the most active catalyst, under the same irradiation conditions. In this analysis, methanol, chloroform, and EDTA were employed to perturb hydroxyl-radical-mediated, oxygen-reduction-related, and hole-mediated pathways, respectively. In the absence of scavengers, MB discoloration reached 86%, whereas the addition of methanol, chloroform, and EDTA reduced it to 11%, 34%, and 76%, respectively (Figure 8). The corresponding UV–Vis spectral profiles are provided in Figure S5 of the Supporting Information, showing the evolution of the characteristic MB absorption band at 664 nm under each inhibited condition. These spectra support the inhibition trend observed in Figure 8. The reductions correspond to activity losses of approximately 87%, 60%, and 12% relative to the uninhibited run. The inhibition sequence methanol > chloroform ≫ EDTA indicates that hydroxyl-radical-mediated oxidation is the most strongly affected pathway during MB discoloration over ZH5G15, while oxygen-reduction-related steps remain relevant but secondary. The limited inhibition produced by EDTA indicates that direct oxidation of MB by valence-band holes is not the dominant pathway under the present conditions. Instead, holes are more likely involved in interfacial reactions that contribute to •OH generation from water or surface hydroxyl groups. In parallel, the inhibition caused by chloroform suggests that electron consumption through O_2_ reduction also contributes to the reaction sequence, although less strongly than the hydroxyl-radical route. This distribution of roles is chemically consistent with MB, whose discoloration is highly sensitive to oxidative attack on the conjugated chromophore and therefore strongly reflects near-surface reactive oxygen species chemistry.^16,60^ A similar predominance of hydroxyl-radical-mediated oxidation over oxygen-reduction-assisted pathways has also been reported for MB discoloration over modified ZnO systems.^16,60^

**Figure 8:**
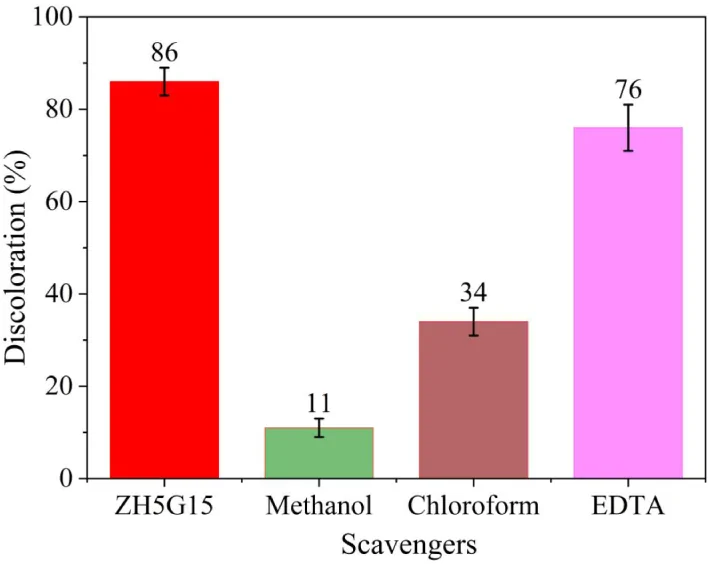
Scavenger tests performed with ZH5G15 during methylene blue (MB) discoloration, using methanol, chloroform, and EDTA as molecular probes for hydroxyl-radical-mediated, oxygen-reduction-related, and hole-mediated pathways, respectively.

Based on these results, the operative sequence for MB discoloration over the Ho/Ga-modified ZnO surface can be described as follows:

3 $$ZH5G15+h\nu \to e_{CB}^{-}+h_{VB}^{+}$$

4 $$e_{CB}^{-}+O_{2}\to O_{2}^{•-}$$

5 $$O_{2}^{•-}+H^{+}⇌{\mathrm{HO}}_{2}^{•}$$

6 $$2{\mathrm{HO}}_{2}^{•}\to H_{2}O_{2}+O_{2}$$

7 $$H_{2}O_{2}+e_{CB}^{-}\to •\mathrm{OH}+{\mathrm{OH}}^{-}$$

8 $$h_{VB}^{+}+H_{2}O\to •\mathrm{OH}+H^{+}$$

9 $$h_{VB}^{+}+OH^{-}\to •\mathrm{OH}$$

10 $$•\mathrm{OH}\left(\mathrm{mainly}\right)+O_{2}^{•-}\left(secondarily\right)+\;MB\to chromophore-disrupted\;MB-derived\;species$$

The scavenger response indicates that the superior MB activity of ZH5G15 is sustained mainly by radical-mediated oxidation at the catalyst surface, with hydroxyl-radical-mediated reactions making the largest contribution and oxygen-reduction-related steps acting as an auxiliary pathway. Under these conditions, codoping primarily affects the efficiency of interfacial ROS generation rather than directly promoting hole-driven oxidation.

Since ZH5G15 showed the highest MB activity, its behavior under repeated operation was subsequently examined. Figure 9a shows that ZH5G15 preserves most of its discoloration capacity over three successive cycles, decreasing only from 86% in the first run to 85%, 84%, and 83% in the subsequent runs. This corresponds to a total loss of about 3.5% relative to the initial activity, indicating that the catalytic response remains largely preserved under the present reaction and recovery conditions. This limited decay indicates that the photocatalytic surface remains largely active over repeated cycles. For a ZnO-based photocatalyst whose performance depends on the balance between charge separation, interfacial transfer, and surface oxidation, maintaining this level of activity after reuse suggests that the codoped framework sustains the operative reaction environment with only limited deterioration during cycling.

**Figure 9:**
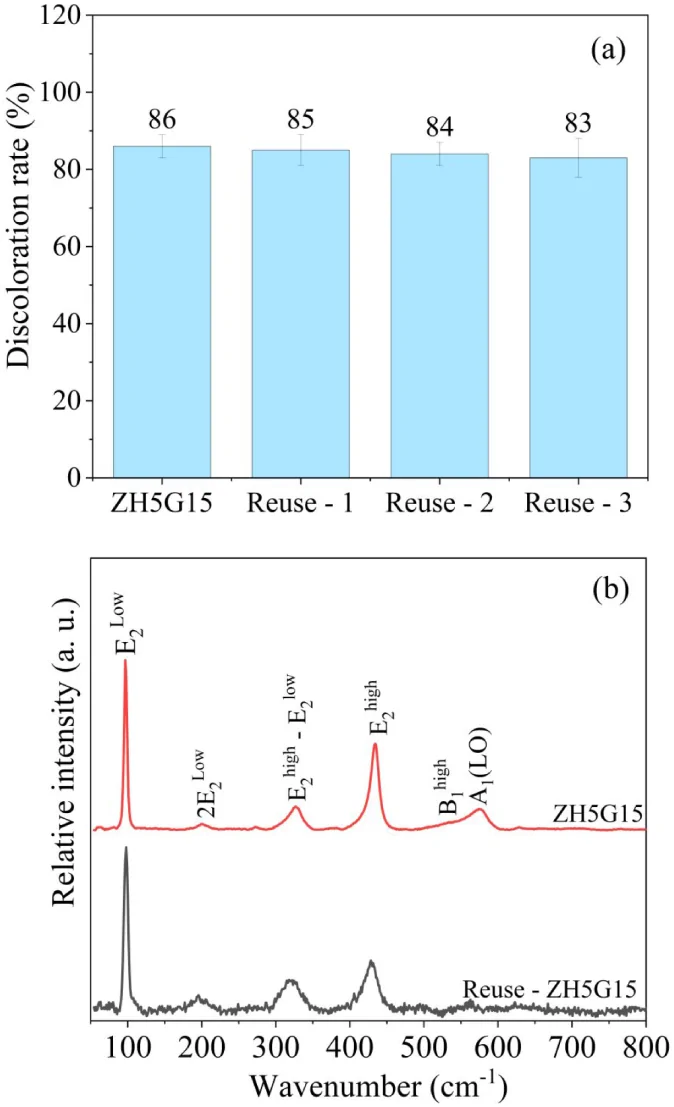
Photocatalytic stability of ZH5G15 evaluated during methylene blue (MB) discoloration: a) reusability performance over three consecutive cycles; b) Raman spectra recorded before reaction and after the reusability tests.

The Raman comparison in Figure 9b supports this preserved activity and compares ZH5G15 before photocatalytic use and after the MB reuse cycles. In both spectra, the characteristic vibrational fingerprint of wurtzite ZnO is maintained, particularly in the E_2_^(low)^, E_2_^(high)^, and LO-related regions. No additional Raman bands attributable to secondary phases or new crystalline species appear after cycling. This indicates that repeated photocatalytic operation does not induce detectable restructuring of the ZnO host lattice. The preservation of the main Raman features, especially the E_2_ modes that are highly sensitive to lattice integrity in wurtzite ZnO,^30^ is consistent with the small loss of MB activity observed during reuse. Thus, the slight decrease in activity is more consistent with minor surface modifications than with structural degradation of the catalyst.

#### 3.4.2 Photocatalytic Removal of Diclofenac

Figure 10a shows the UV–Vis absorbance of the DCF solution as a function of wavelength during irradiation in the presence of ZH5G15. The absorption band centered near 280 nm decreases progressively with time, together with the overall intensity in the 240–300 nm region, indicating the progressive decrease of the parent DCF absorption signal under irradiation. The concentration profiles and final efficiencies in Figure 10b, c show that DCF removal also depends strongly on composition. Because the dark stage leaves C/C_0_ essentially unchanged, the differences observed among the samples arise predominantly under irradiation rather than from prior uptake at the solid–liquid interface. After 120 min, photolysis accounts for only about 13% DCF removal, whereas the catalytic runs reach about 58% for ZH0G0, 70% for ZH5G15, and 50% for ZH2G3. The higher photolysis observed for DCF arises from its greater intrinsic photolability under UV irradiation, as its chlorinated aromatic pharmaceutical structure is more prone to direct photoinduced transformation than the MB chromophore, whose discoloration under catalyst-free conditions remains limited due to the lack of catalyst-mediated ROS formation. Under catalytic conditions, ZH5G15 exceeds pristine ZnO by about 21% and ZH2G3 by about 40%. These differences reflect variations in photo-oxidative efficiency under irradiation.^56,57^ The maximum activity at the intermediate composition indicates that moderate Ho/Ga codoping is consistent with a more favorable condition for interfacial oxidation, whereas the stronger structural perturbation accumulated in ZH2G3 does not translate into more effective use of photogenerated carriers. A similar behavior was reported for the Pr/Al codoped ZnO system under UV irradiation, in which the best composition reached 71.42% DCF removal, and the activity was likewise correlated with structural distortion and defect redistribution.^61^

**Figure 10:**
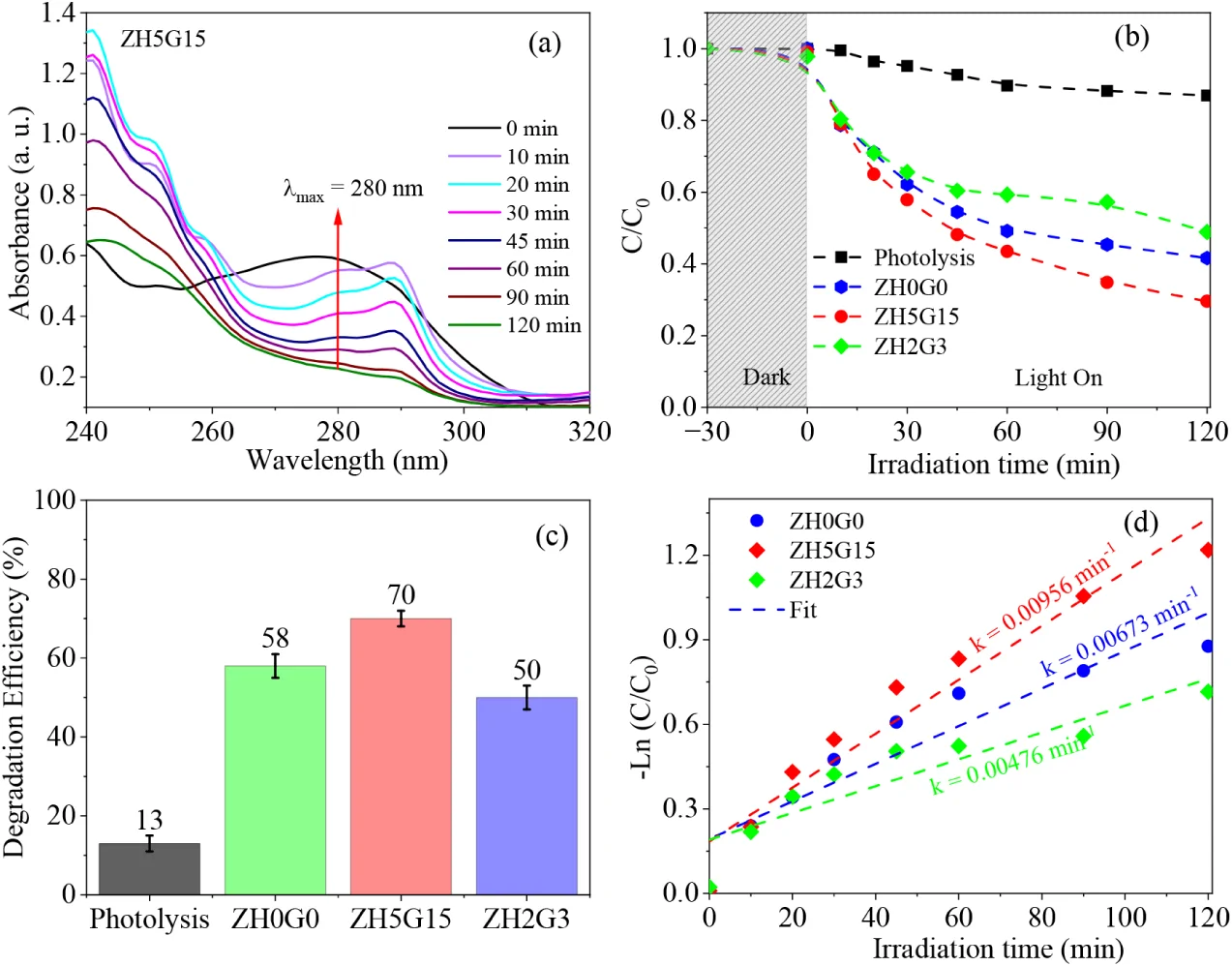
Photocatalytic removal of diclofenac (DCF). a) UV–Vis spectral evolution of DCF over ZH5G15; b) normalized concentration (C/C_0_) versus time for photolysis and catalytic systems; c) removal efficiencies after 120 min for photolysis, ZH0G0, ZH5G15, and ZH2G3; and d) pseudo-first-order kinetic plots (−ln­(C/C_0_) vs time) with fitted rate constants (k).

Figure 10d shows that the kinetic behavior reproduces the activity sequence observed from the concentration profiles and final DCF removal. Under the present conditions, the plots of −ln­(C/C_0_) versus irradiation time are reasonably fitted by an apparent pseudo-first-order model.

The corresponding rate constants are 0.00476 min^–1^ for ZH2G3, 0.00673 min^–1^ for ZH0G0, and 0.00956 min^–1^ for ZH5G15. Accordingly, ZH5G15 decreases the DCF absorption signal 1.42 times faster than pristine ZnO and 2.01 times faster than ZH2G3. This indicates that the kinetic advantage of ZH5G15 is maintained throughout the reaction. A comparative summary of recently reported doped and codoped ZnO-based photocatalysts for MB and DCF removal is provided in Table S1. Despite differences in irradiation conditions, pollutant concentration, catalyst loading, and reaction time, ZH5G15 shows competitive apparent rate constants for both pollutants, particularly considering that the same composition was active toward a dye and a pharmaceutical contaminant under the same experimental configuration. Since DCF adsorption is negligible under the present conditions, the higher activity of ZH5G15 is consistent with a greater ability to sustain oxidative conversion in the liquid phase. Under these conditions, DCF oxidative conversion depends primarily on the generation and persistence of reactive oxidizing species in solutions. Within this series, ZH5G15 provides the most effective condition for DCF conversion: the structural modification is sufficient to alter the optical and interfacial response of ZnO, but not so extensive that additional disorder compromises the photocatalytic trend. In contrast, the higher codoping level in ZH2G3 increases disorder without improving oxidative conversion. This distinction is relevant because the observed activity depends on how effectively irradiation promotes reactive oxidizing species in solution, in agreement with the defect-related PL redistribution and near-edge broadening observed after Ho/Ga incorporation.

Co-doping redistributes the defect-assisted emission and perturbs the near-edge absorption region, indicating that the electronic landscape of ZnO is modified after Ho/Ga incorporation. In ZH5G15, however, this modification remains within a range that still favors productive oxidative conversion, whereas the stronger perturbation accumulated in ZH2G3 does not provide a comparable gain in DCF removal or reaction rate. Thus, the lower performance of ZH2G3 indicates that excessive disorder does not improve the conversion of photogenerated carriers into oxidative processes.

To clarify which oxidative pathways sustain DCF removal over ZH5G15, scavenger tests were performed under the same irradiation conditions used in Figure 10. In this case, methanol, chloroform, and EDTA were used to perturb hydroxyl-radical-mediated, oxygen-reduction-related, and hole-mediated pathways, respectively. In the absence of scavengers, DCF removal reached 70%, whereas the addition of methanol, chloroform, and EDTA decreased efficiency to 56%, 60%, and 62%, respectively (Figure 11). These changes correspond to decreases of 14, 10, and 8 percentage points relative to the uninhibited run. Methanol produced the largest suppression, suggesting that hydroxyl-radical-mediated oxidation is the most influential route under the present conditions. However, the comparatively small separation between the chloroform and EDTA runs indicates that oxygen-reduction-related steps and photogenerated holes remain relevant within the reaction network. Accordingly, DCF oxidative conversion is better described as a cooperative process in which •OH-mediated reactions make the largest contribution, while electron-mediated oxygen activation and hole-assisted interfacial reactions also participate in parallel.

**Figure 11:**
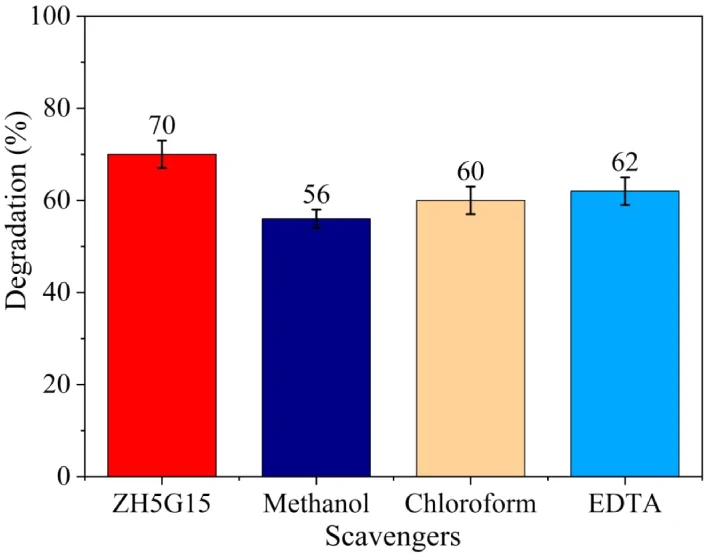
Scavenger tests performed with ZH5G15 during DCF removal, using methanol, chloroform, and EDTA as molecular probes for hydroxyl-radical-mediated, oxygen-reduction-related, and hole-mediated pathways, respectively.

This interpretation is consistent with the negligible adsorption of DCF under the present conditions, which favors oxidation in the liquid phase rather than surface-mediated removal. Hydroxyl radicals play a key role due to their high redox potential (E ∼ 2.8 V vs NHE), enabling nonselective attack on the aromatic structure, including hydroxylation, dechlorination, and bond cleavage.^17,61,62^ In contrast, oxygen-reduction-derived species, although generally less oxidative than •OH, can participate in electron-transfer processes and act as precursors for secondary oxidizing species such as H_2_O_2_, which may further contribute to •OH generation. At the same time, valence-band holes can oxidize surface hydroxyl groups or water, indirectly sustaining •OH formation rather than acting primarily through direct oxidation of DCF.^17,61–63^

The effects of chloroform and EDTA indicate that oxygen activation by photogenerated electrons and hole-assisted interfacial reactions are both coupled to the overall ROS chemistry. Thus, the results are more consistent with a coupled redox scheme than with a mechanism controlled by a single reactive species. Based on this response, the operational sequence for DCF removal over the Ho/Ga-modified ZnO surface can be described as follows:

11 $$ZH5G15+h\nu \to e_{CB}^{-}+h_{VB}^{+}$$

12 $$e_{CB}^{-}+O_{2}\to O_{2}^{•-}$$

13 $$O_{2}^{•-}+H^{+}⇌{\mathrm{HO}}_{2}^{•}$$

14 $$2{\mathrm{HO}}_{2}^{•}\to H_{2}O_{2}+O_{2}$$

15 $$H_{2}O_{2}+e_{CB}^{-}\to •\mathrm{OH}+{\mathrm{OH}}^{-}$$

16 $$h_{VB}^{+}+H_{2}O\to •\mathrm{OH}+H^{+}$$

17 $$h_{VB}^{+}+OH^{-}\to •\mathrm{OH}$$

18 $$•\mathrm{OH}+O_{2}^{•-}+h+DCF+ROS\to partially\;oxidized\;DCF-derived\;species/products$$

This reaction sequence follows the same fundamental ROS-generation steps commonly considered for ZnO-based photocatalysts, including those invoked for methylene blue discoloration.^17,61–63^ However, in the case of DCF, the relative contribution of each pathway is redistributed because adsorption is negligible and the oxidation proceeds under a more solution-driven regime. Within this scheme, the scavenger response indicates that the improved DCF activity of ZH5G15 is sustained by a surface redox environment able to generate multiple oxidizing species under irradiation, rather than by a single exclusive pathway. This interpretation is consistent with the intermediate codoping level, which provides sufficient electronic and defect-related modification to promote ROS formation without the stronger perturbation observed for ZH2G3.

The reuse behavior of ZH5G15 during DCF removal is shown in Figure 12a. The photocatalytic efficiency decreases from 70% in the first cycle to 68%, 60%, 58%, and 53% in the subsequent runs, corresponding to a cumulative loss of 17 percentage points after the fourth cycle. Expressed relative to the initial performance, the catalyst retains approximately 97%, 86%, 83%, and 76% of its original activity from the first to the fourth reuse cycle. These results indicate that ZH5G15 remains active after repeated operation, but the progressive decay also shows that the active reaction environment is not fully preserved during cycling. Compared with MB, the more pronounced decay observed for DCF reflects the different reaction regime, which is more sensitive to interfacial changes affecting ROS generation.

**Figure 12:**
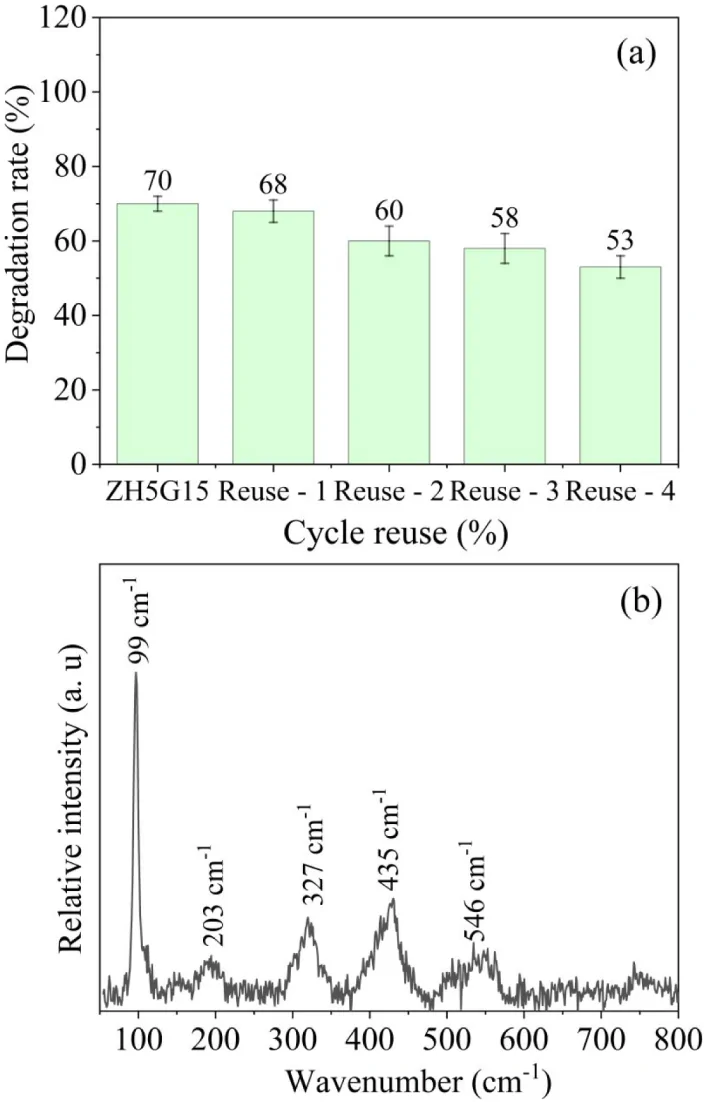
Photocatalytic stability of ZH5G15 during DCF removal: a) reusability over four consecutive cycles; b) Raman spectra recorded before reaction and after reuse, used to assess structural stability.

The Raman spectrum obtained after the reuse cycles (Figure 12b) helps clarify the nature of this deactivation. The main vibrational features remain in the same spectral regions observed for the fresh material, including the characteristic ZnO modes near 99, 327, 435, and 546 cm^–1^. No additional bands attributable to new crystalline phases are observed, and no marked spectral changes suggest a major reconstruction of the ZnO host lattice.

Within the detection limit of Raman spectroscopy, these results indicate that repeated photocatalytic operation does not induce structural collapse or phase transformation of the codoped ZnO framework. Accordingly, the decrease in activity is unlikely to arise from degradation of the bulk crystalline framework. A similar use of Raman preservation as evidence of structural persistence after photocatalytic cycling has been reported for ZnO-based systems and other oxide photocatalysts, where activity loss was associated more strongly with surface effects than with bulk crystallographic instability.^30^ Therefore, the decrease in DCF removal is more reasonably associated with interfacial deactivation processes than with loss of the wurtzite structure.

One plausible contribution is the progressive modification of the catalyst/solution interface during DCF oxidation. In photocatalytic systems involving aromatic pharmaceuticals, hydroxylation, dechlorination, and ring-opening reactions can generate organic species with different polarity, adsorption affinity, and surface residence time compared with the parent molecule.^56,62^ Their partial retention at active interfacial sites may hinder the access of water, hydroxyl groups, and dissolved oxygen to the catalyst surface, thereby weakening the electron-transfer and ROS-generation steps required to sustain DCF conversion. In a system such as ZH5G15, where the scavenger tests indicate that hydroxyl-radical-mediated oxidation is the most influential route while oxygen-reduction-related and hole-mediated steps also contribute, this interfacial modification is especially relevant because it can simultaneously affect oxygen activation, hole-mediated reactions, and radical formation.

A second likely contribution is the gradual modification of the most reactive surface defect environment during repeated irradiation and recovery steps. The superior activity of ZH5G15 was previously correlated with an intermediate degree of lattice perturbation, redistributed defect-assisted optical response, and the highest accessible surface area in the series. Thus, its reuse behavior depends not only on preserving the ZnO lattice but also on maintaining the interfacial sites involved in ROS formation. Even if the crystalline and vibrational framework remains largely intact, repeated use may change the surface population of hydroxyl groups, adsorbed oxygen species, or defect-related reactive sites that directly participate in ROS formation. In this sense, the Raman result does not indicate major structural deterioration, but it does not exclude more subtle chemical or electronic changes at the outermost surface, which may still be sufficient to reduce photocatalytic performance over reused cycles.

The progressive character of deactivation also deserves attention. The drop is small between the first and second cycles, but becomes more evident from the third run onward. This suggests that deactivation is cumulative rather than abrupt, which is more compatible with gradual surface fouling, incomplete removal of surface-retained organic residues during catalyst recovery, or progressive loss of the most reactive interfacial sites than with sudden structural failure. The fact that ZH5G15 still preserves 53% DCF removal after the fourth cycle indicates that the catalyst remains active, although under a less favorable surface chemical state than in the fresh catalyst.

## 4 Conclusion

In summary, Zn_1–x–y_Ho_x_Ga_y_O powders were obtained by coprecipitation and preserved the wurtzite ZnO framework after calcination at 450 °C. Ho^3+^/Ga^3+^ codoping did not yield a monotonic improvement in the photocatalytic response of ZnO with increasing dopant content. Instead, the highest activity occurred at the intermediate composition, where structural modification, defect-related optical changes, and surface accessibility evolved in ways more favorable to photocatalysis. Within this structural framework, codoping induced a slight lattice contraction, progressive crystallite refinement, higher microstrain, local lattice perturbation, and selective changes in defect-related optical states. Among the investigated samples, Zn_0.98_Ho_0.005_Ga_0.015_O combined controlled structural modification with the highest accessible surface area, reaching 12.4 m^2^ g^–1^, whereas the more heavily codoped composition accumulated additional disorder without improving the photocatalytic response. Accordingly, Zn_0.98_Ho_0.005_Ga_0.015_O exhibited the highest activity toward both probe contaminants, with removal efficiencies of 86% for methylene blue and 70% for diclofenac after 120 min, together with the highest apparent rate constants in the series. The comparison between methylene blue and diclofenac further showed that the same photocatalyst responds under two distinct interfacial regimes, one more influenced by surface-mediated processes and the other more dependent on oxidation in the liquid phase. Scavenger tests showed that hydroxyl-radical-mediated oxidation was the most strongly affected pathway, while oxygen-reduction-related and hole-mediated steps made secondary but relevant contributions depending on the contaminant. Reuse experiments, together with the postreaction Raman results, indicate that the loss of activity is more consistently associated with interfacial deactivation than with structural collapse of the ZnO host. Within the investigated Zn_1–x–y_Ho_x_Ga_y_O series, the best photocatalytic performance was achieved for the moderate Ho/Ga incorporation regime, where oxidative conversion at the catalyst interface was favored while avoiding the stronger lattice disorder associated with higher codoping levels. This result highlights the importance of controlling codopant content to tune ZnO photocatalysts for pollutant-dependent oxidation processes.

## Supplementary Material



## Data Availability

All the data required to reproduce these experiments are present in the article.

## Notes

Baseline-corrected Raman spectra and oscillator-function fitting results for ZH0G0, ZH5G15, and ZH2G3, including fitted components and residual curves; EDS spectra of the undoped and Ho/Ga codoped ZnO samples, providing complementary qualitative compositional information, as well as a schematic energy-level representation of the Gaussian-deconvoluted PL components within the ZnO band gap range; UV–Vis absorption spectra of methylene blue recorded during scavenger-assisted photocatalytic discoloration tests over ZH5G15 are also provided to support the discussion of the reactive species pathways; Urbach plots used to estimate the absorption-tail energies and a benchmarking table comparing the apparent rate constants of ZH5G15 with recently reported ZnO-based photocatalysts for MB and DCF removal (PDF)
