# Supplementary material for: Enhanced Methylene Blue and Diclofenac Removal in Ho/Ga Codoped ZnO: Balancing Defect Chemistry, Surface Accessibility, and Interfacial Oxidation
Source: ACS Omega. 2026 Aug 18;11(34):51226–42. doi: 10.1021/acsomega.6c04116 (PMC13625088; doi:10.1021/acsomega.6c04116)
Supplement: Supplementary file 1 [file ao6c04116_si_001.pdf]

Supplementary information

For

Enhanced methylene blue and diclofenac removal in Ho/Ga co-doped ZnO: balancing defect chemistry, surface accessibility, and interfacial oxidation

Yonny Romaguera-Barcelay[a,*], Ellyson S. Paula-Alves[a], Aimée G. Jerônimo[b], Willams A. S. Albuquerque, Luciano C. Almeida[c] Ramón R. Peña-Garcia[a,b**].

[a]Universidade Federal Rural de Pernambuco, Programa de pós-graduação em Engenharia Física, Unidade Acadêmica do Cabo de Santo Agostinho, Cabo de Santo Agostinho, PE, Brasil.

[b]Universidade Federal de Piauí, Pós-graduação em Ciências e Engenharia dos Materiais, Teresina, PI, Brasil.

[c]Universidade Federal de Pernambuco, Departamento de Engenharia Química, Recife-PE, Brasil.

**Corresponding author: E-mail: ramon.raudel@ufrpe.br, rraudelp@gmail.com and yonnyroma@gmail.com*

1. Raman spectroscopy

Raman spectroscopy confirmed the preservation of the ZnO wurtzite structure and indicated that Ho^{3+}/Ga^{3+} incorporation induced subtle local lattice perturbations. Variations in band shape and broadening suggest defect redistribution and short-range structural distortion in the co-doped samples. **Figure S1a-c** shows the baseline-corrected Raman spectra and oscillator-function fits, including the fitted components and residuals, for the samples

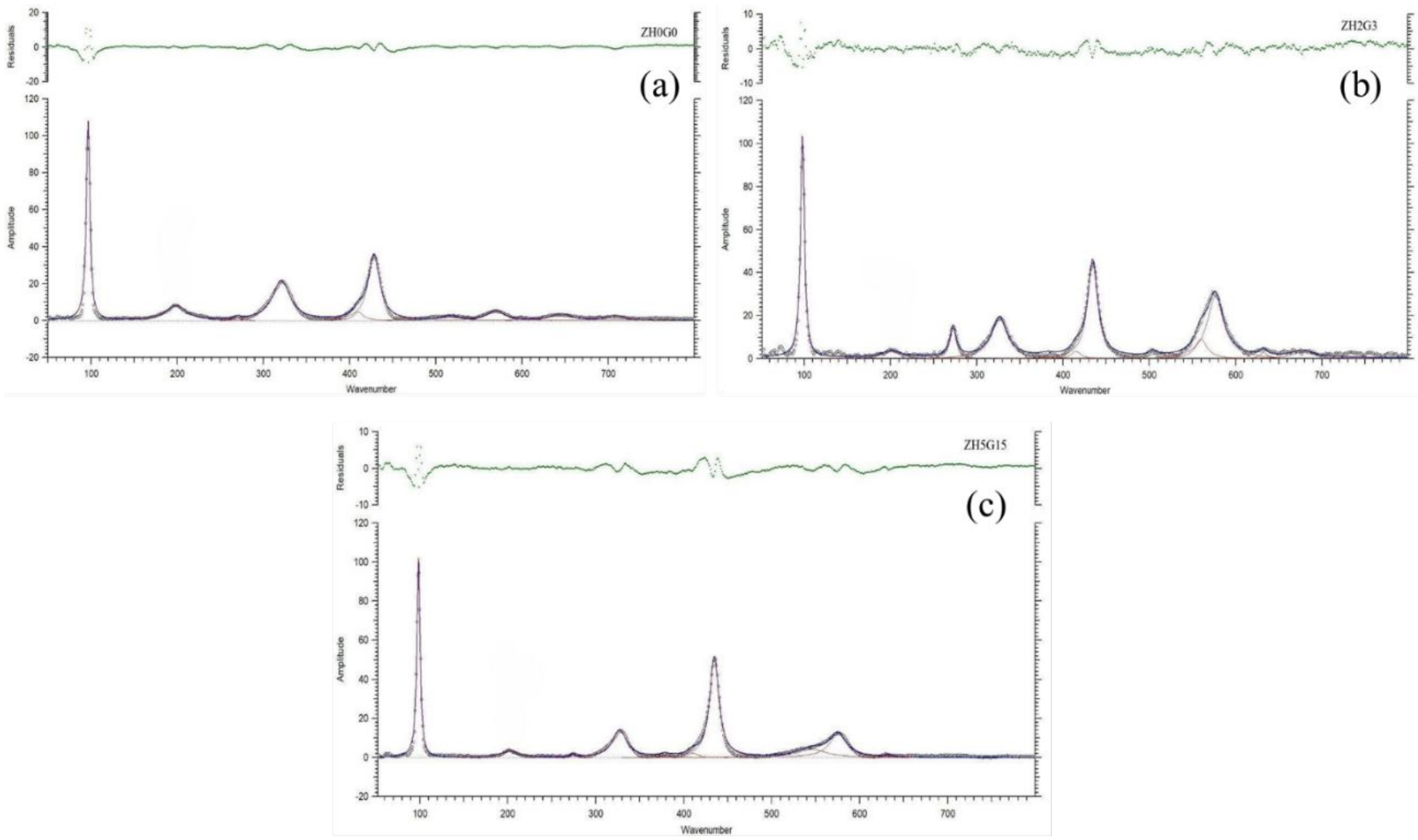

Figure S1. Baseline-corrected Raman spectrum and oscillator-function fit (including residual and fitted components) for a) ZH0G0, b) ZH5G15, and c) ZH2G3.

2. EDS spectra of undoped and Ho/Ga co-doped ZnO samples.

The EDS spectra shown in Figure S2 provide complementary qualitative compositional information for the regions analyzed by SEM/EDS mapping. The ZH0G0 sample exhibits only the expected Zn and O signals, consistent with the nominal composition of undoped ZnO. In contrast, the co-doped samples ZH5G15 and ZH2G3 show additional Ho- and Ga-related contributions, indicating the presence of the co-dopant elements within the analyzed regions. The Ga-related signal was interpreted qualitatively because of its partial spectral overlap with Zn emissions, and therefore, the spectra were not used for quantitative compositional determination. The Au signal observed in all spectra originates from the conductive coating applied before SEM/EDS analysis.

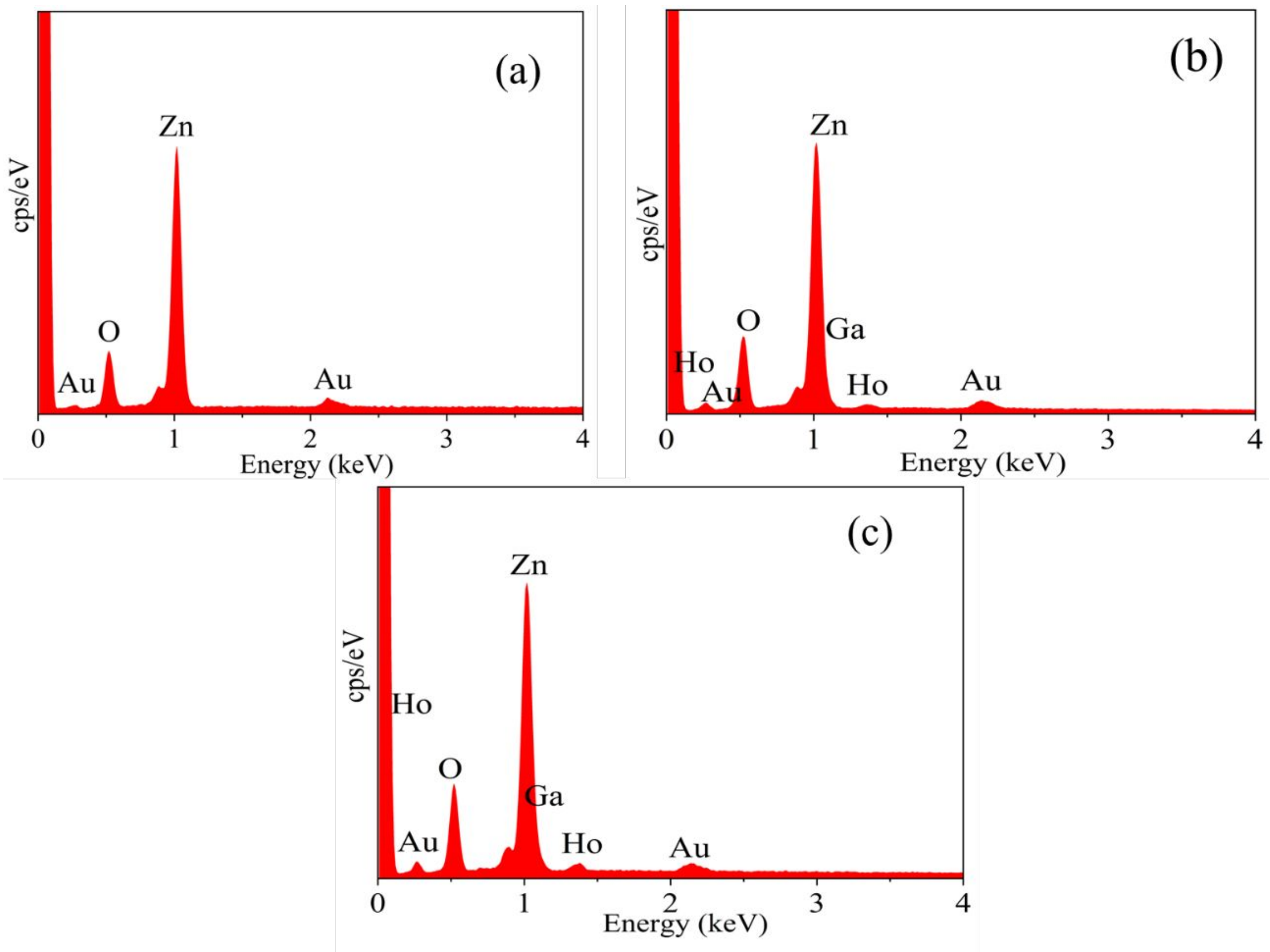

Figure S2. EDS spectra of (a) ZH0G0, (b) ZH5G15, and (c) ZH2G3 samples. The spectra show the expected Zn and O signals for ZH0G0 and additional Ho- and Ga-related contributions for the co-doped samples. The Au signal originates from the conductive coating used during SEM/EDS analysis.

3. Photoluminescence (PL)

Figure S3 visualizes the fitted PL components by converting the Gaussian centers (λ_0) into photon energies using E (eV) = 1240/λ_0 (nm) and positioning them within the measured band gap of each sample.. Across ZH0G0, ZH5G15, and ZH2G3, the diagram highlights the redistribution of visible-emission components across the blue-to-red spectral range, while the optical band gap remains nearly unchanged. This representation is intended to illustrate relative spectral trends rather than to assign each component uniquely to a specific defect, since visible photoluminescence in ZnO is well known to consist of broad, overlapping emissions arising from multiple native and surface-related

states, and the unambiguous attribution of individual bands remains debated in the literature [1–3]

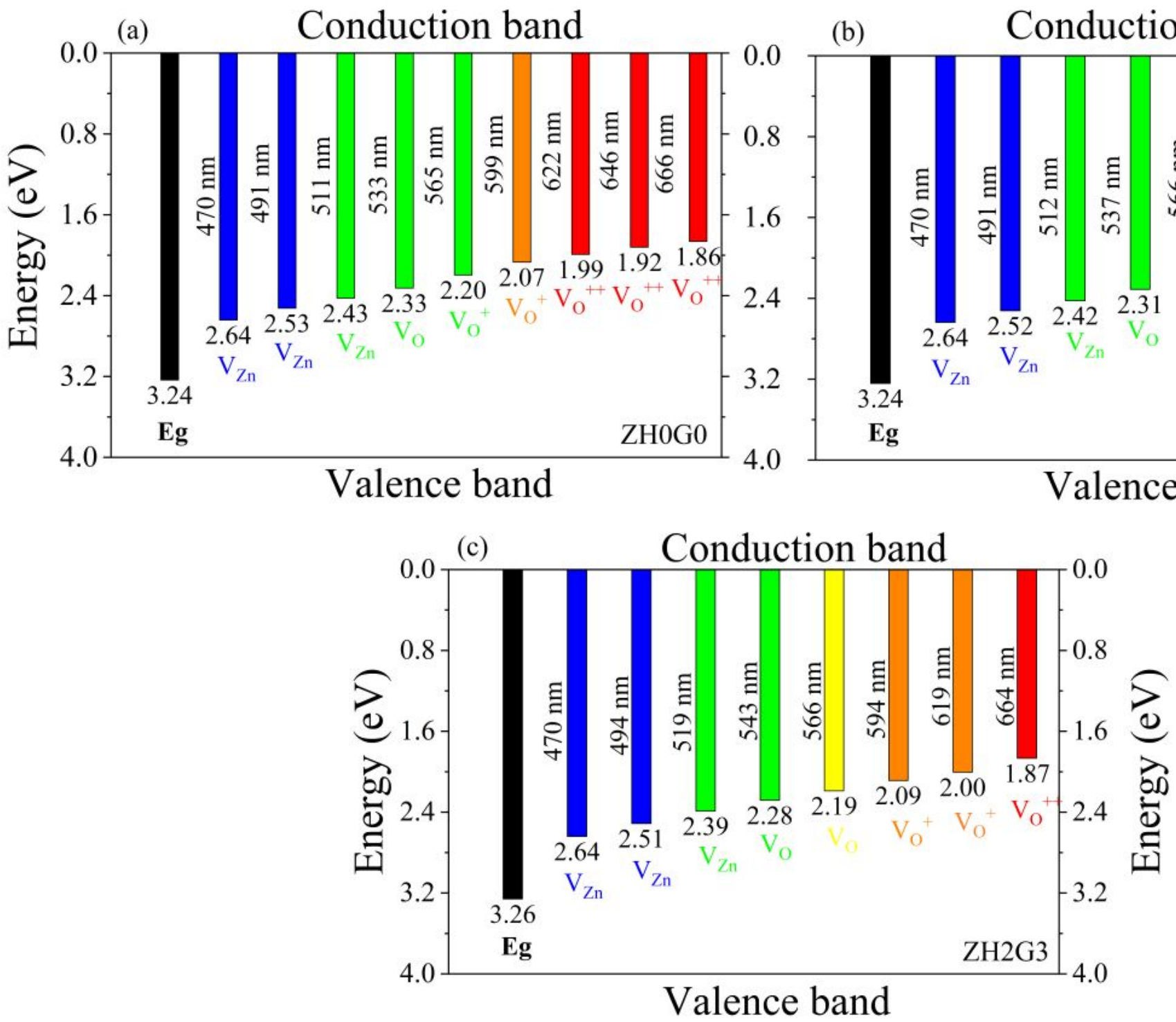

Figure S3. Schematic energy-level diagram of ZnO showing defect-related radiative transitions inferred from the fitted PL peak positions for $Zn_{1-x-y}Ho_xGa_yO$ samples. (a) ZH0G0, (b) ZH5G15, and (c) ZH2G3.

4. Urbach energy calculation

Figure S4 shows the procedure used to estimate the Urbach energy of the samples. After transforming the diffuse-reflectance data through the Kubelka–Munk function, the exponential tail near the absorption edge was identified and its linear region was fitted. The $E_U$values were then obtained from the inverse slope of the fitted lines. Since the Urbach energy is associated with the width of localized tail states, higher $E_U$values indicate a greater degree of structural disorder and defect-related electronic states in the semiconductor.

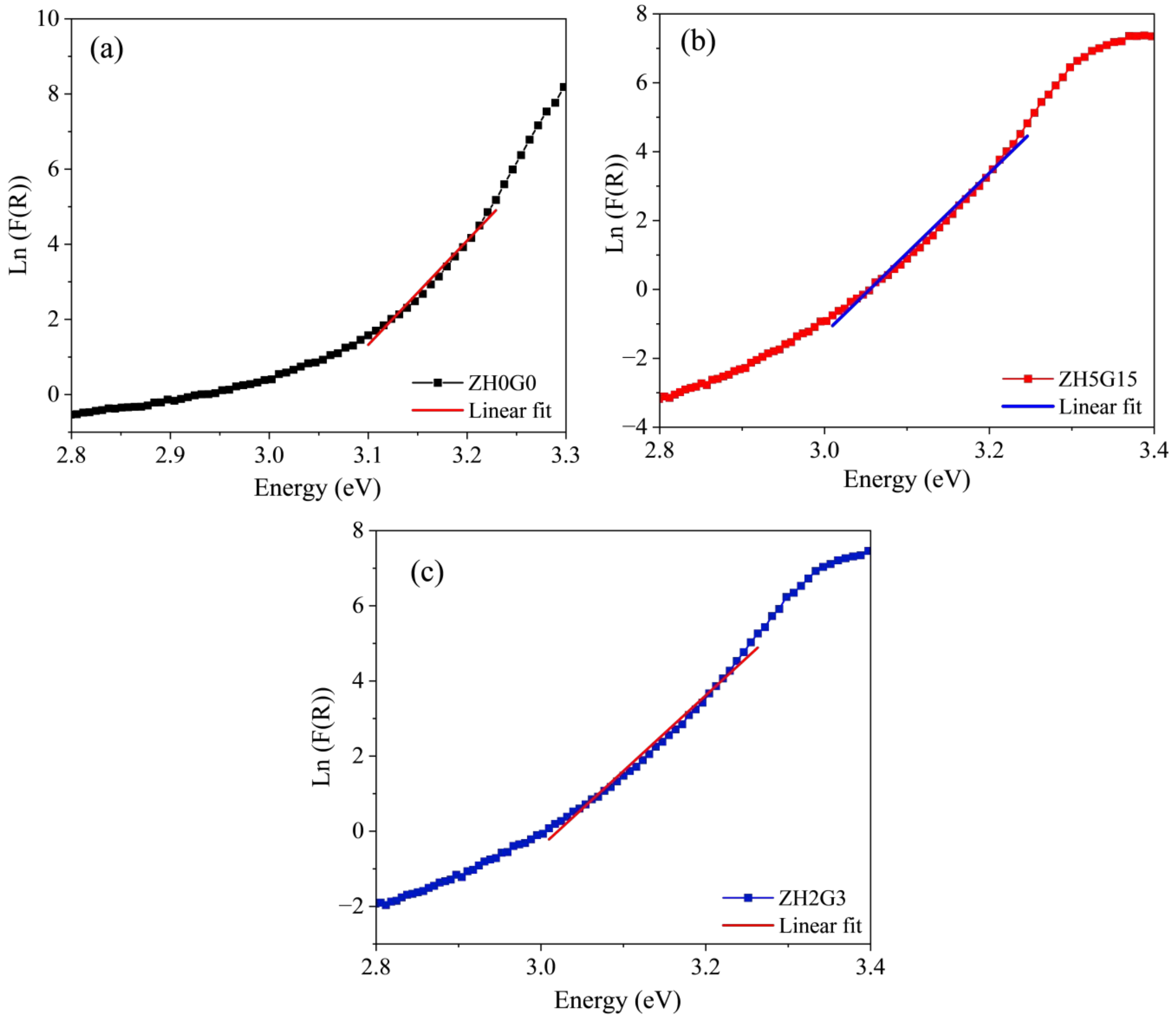

Figure S4. Representative Urbach plots used for determining the Urbach energy (EU) of the ZnO-based samples from Kubelka–Munk-transformed diffuse-reflectance spectra. (a) ZH0G0, (b) ZH5G15, and (c) ZH2G3. Linear fitting of the exponential absorption tail was used to estimate EU from the reciprocal of the slope.

5. UV–Vis spectra of MB degradation over ZH5G15 in the presence of scavengers

Figure S5 shows the UV–Vis absorption spectra of methylene blue (MB) during photocatalytic degradation over ZH5G15 in the presence of different scavengers. Methanol, chloroform, and EDTA were used to probe the contributions of •OH radicals, O_2•$^-$-related pathways, and photogenerated holes (h^+), respectively. In the presence of methanol, the characteristic MB absorption band at 664 nm remains largely preserved

after irradiation, indicating strong inhibition of MB discoloration when •OH radicals are scavenged. Chloroform produces an intermediate decrease in the 664 nm band, suggesting that $O_2•^-$-related pathways also contribute to the degradation process. In contrast, EDTA leads to a more pronounced decrease in the MB absorption band, indicating that direct h^+-mediated oxidation is less dominant under the present conditions. Overall, these spectral changes support the scavenger results discussed in the main text and indicate that MB discoloration over ZH5G15 is mainly governed by hydroxyl-radical-mediated oxidation, with secondary participation of $O_2•^-$-related pathways.

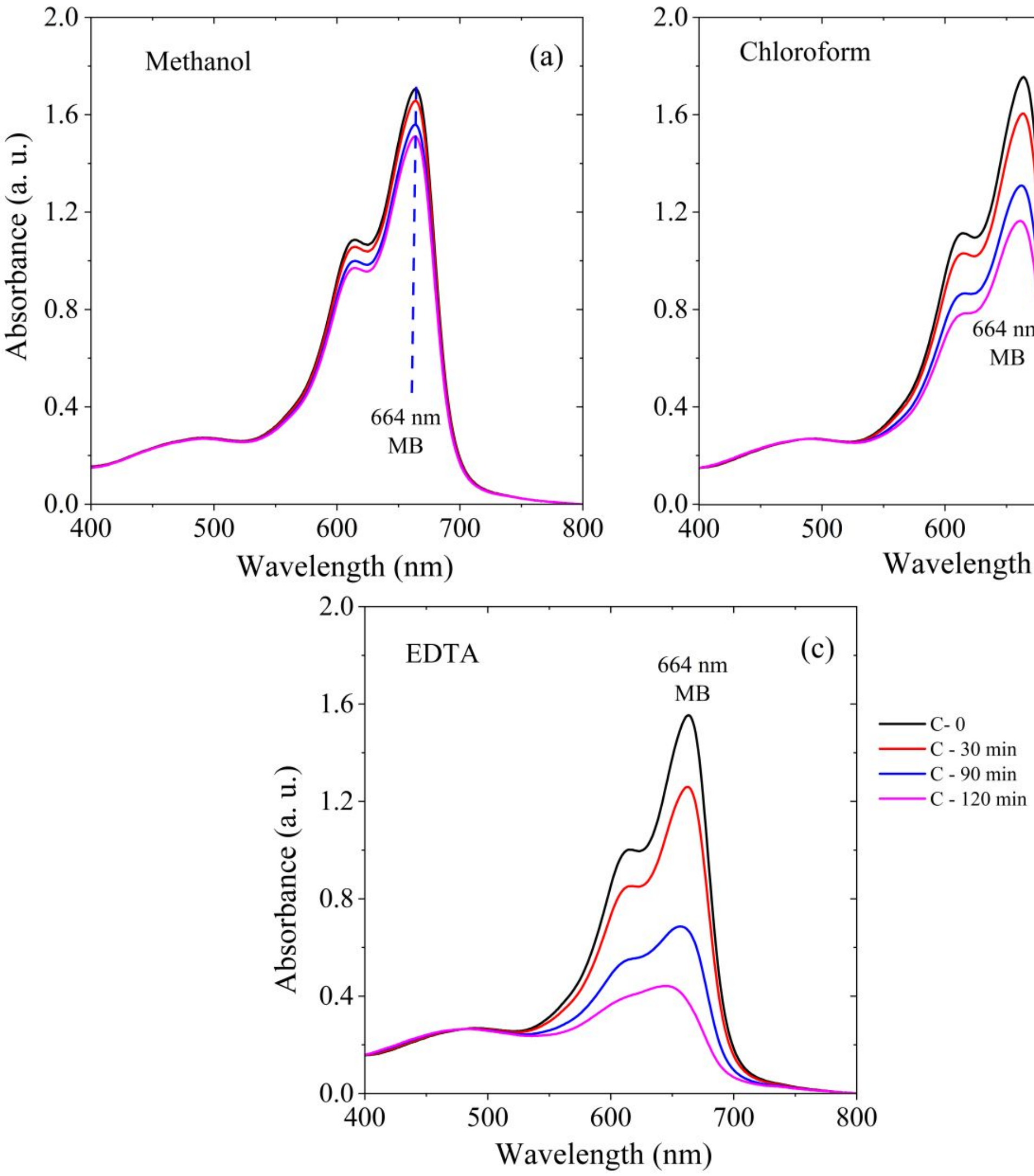

Figure S5. UV–Vis absorption spectra of methylene blue (MB) during photocatalytic degradation over ZH5G15 in the presence of different scavengers: (a) methanol, used as

a •OH scavenger; (b) chloroform, used as a probe for $O_2^{\bullet -}$-related pathways; and (c) EDTA, used as an h^+ scavenger. The evolution of the characteristic MB absorption band at 664 nm supports the predominant role of •OH radicals and the secondary contribution of $O_2^{\bullet -}$-related pathways during MB discoloration.

Table S1. Benchmarking of the photocatalytic performance of ZH5G15 against recently reported ZnO-based photocatalysts for MB and DCF removal.

**Entry**	**Photocatalyst**	**Pollutant**	**Irradiation conditions**	**Removal efficiency (%)**	**kapp (min^{-1})**	**Reusability**	**Ref.**
1	**ZH5G15, $Zn_{0.98}Ho_{0.005}Ga_{0.015}O$**	MB	UV, 125 W; 350-450 nm	**86.0% / 120 min**	**0.0161**	3 cycles: 86% -> 83%	This work
2	NiO-decorated ZnO nanowhiskers	MB	UV, 365 nm, 8 W	72% / 80 min	0.0150	Reused once: 72% -> 66%; good short-term stability	[4]
3	10% Cl-doped ZnO, pH 11	MB	UV light	81.19% / 90 min	0.0150	Recycling not reported	[5]
4	10% Cl-doped ZnO, pH 5	MB	UV light	71.35% / 90 min	0.0140	Recycling not reported	[5]
5	5% Cl-doped ZnO, pH 11	MB	UV light	75.20% / 90 min	0.0120	Recycling not reported	[5]
6	Ho-doped ZnO, 5% Ho	MB	Visible light	83.95% / 150 min	0.0115	Recycling not reported	[6]
7	ZnO, sol-gel	MB	UV light	86% / 140 min	0.0106	Low-cost sol-gel route; reuse NR	[7]
8	5% Cl-doped ZnO, pH 5	MB	UV light	64.47% / 90 min	0.0100	Recycling not reported	[5]

11	ZnO nanowhiskers	MB	UV, 365 nm, 8 W	54% / 80 min	0.0090	NR	[4]
12	NiO-ZnO nanocomposite	MB	UV, 365 nm, 8 W	66% / 80 min after reuse	0.0130	Recycled NiO-ZnO sample	[4]
14	Co-doped ZnO thin film, 5% Co	MB	Visible irradiation	62.6% / 140 min	0.0072	Immobilized thin film; recycling not reported	[8]
15	ZnO, pristine sample from Gd-ZnO series	MB	UV light / Xe lamp, 500 W	40% / 120 min	0.0065	NR	[9]
16	Co-doped ZnO thin film, 3% Co	MB	Visible irradiation	45.7% / 140 min	0.0042	Immobilized thin film; recycling not reported	[8]
17	Cu-doped ZnO thin film, 5% Cu	MB	Visible irradiation	42.5% / 140 min	0.0040	Immobilized thin film; recycling not reported	[8]
18	Cu-doped ZnO thin film, 1% Cu	MB	Visible irradiation	36.2% / 140 min	0.0034	Immobilized thin film; recycling not reported	[8]
20	Cu-doped ZnO thin film, 3% Cu	MB	Visible irradiation	37.7% / 140 min	0.0034	Immobilized thin film; recycling not reported	[8]
21	Co-doped ZnO thin film, 1% Co	MB	Visible irradiation	30.4% / 140 min	0.0026	Immobilized thin film; recycling not reported	[8]
22	Ti-doped ZnO	MB	UV irradiation	NR	0.00254	Recycling not reported	[10]

23	Ho-doped ZnO series, 0% Ho / ZnO	MB	Visible light	NR	0.00201	Recycling not reported	[6]
24	ZnO from Ti-ZnO study	MB	UV irradiation	NR	0.00140	Recycling not reported	[10]
25	ZnO thin film	MB	Visible irradiation	2.7% / 140 min	0.0002	Immobilized thin film; recycling not reported	[8]
1	**ZH5G15, $Zn_{0.98}Ho_{0.005}Ga_{0.015}O$**	DCF	UV, 125 W; 350–450 nm	**70% / 120 min**	**0.00956**	4 cycles; activity retained	This work
3	ZnO nanoparticles	DCF	Visible-light photocatalysis	48.9% / 120 min	0.00594	NR for ZnO control	[11]
4	$Zn_{1-x-y}Pr_xAl_yO$, ZPA31	DCF	UV photocatalysis	NR	0.00725	Recyclability reported for the series	[12]
5	$Zn_{1-x-y}Pr_xAl_yO$, ZPA33	DCF	UV photocatalysis	Up to 71.42%	0.00798	Highly recyclable/stable according to abstract	[12]
6	$ZnFe_2O_4$ nanoparticles	DCF	Visible-light photocatalysis	61.4% / 120 min	0.00883	Stable up to five cycles; 3.9% loss	[11]

References

[1] A. Mahesh, I.N. Jawahar, V. Biju, Photoluminescence, photocurrent response and photocatalytic activity of hydrothermally derived nanocrystalline ZnO with native point defects, J. Cryst. Growth 648 (2024) 127894. https://doi.org/10.1016/j.jcrysgro.2024.127894.

[2] H. Rai, Prashant, N. Kondal, A review on defect related emissions in undoped ZnO nanostructures, Mater. Today Proc. 48 (2022) 1320–1324. https://doi.org/10.1016/j.matpr.2021.08.343.

[3] B.J. Alotibi, A. Loucif, A. Majid, Tailoring the Optical Characteristics of ZnO Nanoparticles by Fe/Co Dual-Doping, Physics of the Solid State 65 (2023) 119–130. https://doi.org/10.1134/S1063783423600413.

[4] I. Abdul Rahman, M.T.M. Ayob, S. Radiman, Enhanced Photocatalytic Performance of NiO-Decorated ZnO Nanowhiskers for Methylene Blue Degradation, J. Nanotechnol. 2014 (2014) 1–8. https://doi.org/10.1155/2014/212694.

[5] R.A. Alshgari, Z.A. Ujjan, A.A. Shah, M.A. Bhatti, A. Tahira, N.M. Shaikh, S. Kumar, M.H. Ibupoto, A. Elhawary, A. Nafady, B. Vigolo, Z.H. Ibhupoto, ZnO Nanostructures Doped with Various Chloride Ion Concentrations for Efficient Photocatalytic Degradation of Methylene Blue in Alkaline and Acidic Media, Molecules 27 (2022) 8726. https://doi.org/10.3390/molecules27248726.

[6] A. Phuruangrat, S. Thamsukho, T. Thongtem, S. Thongtem, Combustion synthesis and analysis of visible-light-driven Ho-doped ZnO photocatalytic nanoparticles, Desalination Water Treat. 273 (2022) 212–220. https://doi.org/10.5004/dwt.2022.28863.

[7] K.A. Isai, V.S. Shrivastava, Photocatalytic degradation of methylene blue using ZnO and 2%Fe–ZnO semiconductor nanomaterials synthesized by sol–gel method: a comparative study, SN Appl. Sci. 1 (2019) 1247. https://doi.org/10.1007/s42452-019-1279-5.

[8] W. Vallejo, A. Cantillo, B. Salazar, C. Diaz-Uribe, W. Ramos, E. Romero, M. Hurtado, Comparative Study of ZnO Thin Films Doped with Transition Metals (Cu

and Co) for Methylene Blue Photodegradation under Visible Irradiation, Catalysts 10 (2020) 528. https://doi.org/10.3390/catal10050528.

[9] A. Alasmari, N. Alresheedi, M. Alzahrani, F. Aldosari, M. Ghasemi, A. Ismail, A. Aboraia, High-Performance Photocatalytic Degradation—A ZnO Nanocomposite Co-Doped with Gd: A Systematic Study, Catalysts 14 (2024) 946. https://doi.org/10.3390/catal14120946.

[10] S. Wongrerkdee, S. Wongrerkdee, C. Boonruang, S. Sujinnapram, Enhanced Photocatalytic Degradation of Methylene Blue Using Ti-Doped ZnO Nanoparticles Synthesized by Rapid Combustion, Toxics 11 (2022) 33. https://doi.org/10.3390/toxics11010033.

[11] S. Latif, A. Liaqat, M. Imran, A. Javaid, N. Hussain, T. Jesionowski, M. Bilal, Development of zinc ferrite nanoparticles with enhanced photocatalytic performance for remediation of environmentally toxic pharmaceutical waste diclofenac sodium from wastewater, Environ. Res. 216 (2023) 114500. https://doi.org/10.1016/j.envres.2022.114500.

[12] E. Martins, P. Trigueiro, A.G. Jerônimo, R. Barbosa, L. Neves, D.A. Sales, L.C. Almeida, B.C. Viana, A.S. Soares, R.R. Peña-Garcia, Efficient photocatalytic degradation of diclofenac drug using the Zn1-x-yPrxAlyO photocatalyst under UV light irradiation, Environmental Science and Pollution Research 31 (2024) 53074–53089. https://doi.org/10.1007/s11356-024-34768-8.
